# Bottom-up design of Ca^2+^ channels from defined selectivity filter geometry

**DOI:** 10.1038/s41586-025-09646-z

**Published:** 2025-10-22

**Authors:** Yulai Liu, Connor Weidle, Ljubica Mihaljević, Joseph L. Watson, Zhe Li, Le Tracy Yu, Sagardip Majumder, Andrew J. Borst, Kenneth D. Carr, Ryan D. Kibler, Tamer M. Gamal El-Din, William A. Catterall, David Baker

**Affiliations:** 1 Department of Biochemistry, University of Washington, Seattle, WA, USA.; 2 Institute for Protein Design, University of Washington, Seattle, WA, USA.; 3 Molecular Engineering and Sciences Institute, University of Washington, Seattle, WA, USA.; 4 Howard Hughes Medical Institute, University of Washington, Seattle, WA, USA.; 5 Department of Pharmacology, University of Washington, Seattle, WA, USA.

## Abstract

Native ion channels play key roles in biological systems, and engineered versions are widely used as chemogenetic tools and in sensing devices^1,2^. Protein design has been harnessed to generate pore-containing transmembrane proteins, but the design of selectivity filters with precise arrangements of amino acid sidechains specific for a target ion, a crucial feature of native ion channels^3^, has been constrained by the lack of methods to place the metal-coordinating residues with atomic-level precision. Here we describe a bottom-up RFdiffusion-based approach to construct Ca^2+^ channels from defined selectivity filter residue geometries, and use this approach to design symmetric oligomeric channels with Ca^2+^ selectivity filters having different coordination numbers and different geometries at the entrance of a wider pore buttressed by multiple transmembrane helices. The designed channel proteins assemble into homogenous pore-containing particles, and for both tetrameric and hexameric ion-coordinating configurations, patch-clamp experiments show that the designed channels have higher conductances for Ca^2+^ than for Na^+^ and other divalent ions (Sr^2+^ and Mg^2+^) that are eliminated upon mutation of selectivity filter residues. Cryo-electron microscopy indicates that the design method has high accuracy: the structure of the hexameric Ca^2+^ channel is nearly identical to the design model. Our bottom-up design approach now enables the testing of hypotheses relating filter geometry to ion selectivity by direct construction, and provides a roadmap for creating selective ion channels for a wide range of applications.

The origin of the ion selectivity of natural channels has been of great interest since the characterization of the first native channels, but experimental approaches to date have been limited to examining the effects of amino acid substitutions on native channel selectivity^4–9^ or domain grafting among homologs to create chimeric channels^10–12^ and have not allowed systematic probing of pore geometry. The ability to construct ion channels from scratch with pre-defined selectivity filters and pore geometries would enable direct testing of hypotheses relating pore structure to conductance, and the creation of custom channels for chemogenetics and sensing applications^1,2,13,14^. Progress has been made in de novo design of pore-containing transmembrane proteins and channels, including assembly of synthetic peptides into barrels^15–19^, conversion of water-soluble helical bundles into transmembrane pores^20,21^, and the design of transmembrane β-barrels from 2D blueprints^22,23^. However, these approaches do not enable precisely placing ion-interacting residues along the ion permeation pathway at angstrom level, and hence designed conducting channels to date have had little ion selectivity. This limitation is particularly significant in the context of designing a Ca^2+^ channel since Ca^2+^ has a nearly identical bare ion radius (~1 Å) to Na^+^ and a hydrated ion radius (~4 Å) similar to Mg^2+ 24–26^. Native Ca^2+^ channels utilize carboxylate groups to bind Ca^2+^ ions with high affinity likely in a partially dehydrated manner contingent on the geometry of the carboxylate groups^27–32^, which prevents permeation of other ions. Such stringent requirements on the geometry of the selectivity filter are not readily achievable using previous design methods, as the positions of pore-lining residues are largely determined by the prior placement of the protein backbone^20–22^.

We sought to develop a general approach for designing ion channels based on ion-coordinating residues that function as the selectivity filter. We chose to focus on Ca^2+^ channels as the mechanism of Ca^2+^ selectivity is relatively well understood, and designed Ca^2+^ channels could serve as useful biological tools due to the critical role of Ca^2+^ as a second messenger. With the hypothesis that the proper spatial arrangement of the carboxyl groups at the pore entrance followed by a wide and well-hydrated pore comprised of residues distant from the axis of the pore would confer both Ca^2+^ permeability and selectivity, we reasoned that Ca^2+^ channels could in principle be constructed by (1) generating selectivity filter configurations by systematically sampling the distance and coordination geometry of carboxylate containing sidechains around a central Ca^2+^ ion, and then using generative deep learning methods to (2) build single helices out from the selectivity filter sidechains that define the overall pore shape, and (3) buttress these central pore helices by multiple surrounding helices. This procedure could in principle create channels with custom-defined selectivity filter geometry with high precision, with the overall topology of the protein generated to support the selectivity filter and conducting pore rather than being defined in advance.

## Selectivity filter generation

All known native Ca^2+^ channels utilize negatively charged Glu or Asp residues in the selectivity filter, but in different configurations with different backbone flexibilities. For example, in Ca^2+^ channels within the superfamily P-loop channels where the Glu/Asp residues are situated on a loop, the voltage-gated Ca^2+^ channel Ca_v_1.1 has the selectivity filter asymmetrically formed by 4 glutamate residues with neighboring oxygen atom distances ranging from 3.3 Å to 5.4 Å^33^, the homotetrameric transient receptor potential vanilloid 6 (TRPV6) channel has four Asp residues with diagonal oxygen atom distances of 4.6 Å^34^, while this same diagonal distance in the Ca_v_ab channel (engineered from a bacterial voltage-gated Na^+^ channel Na_v_ab) is 8 Å^5^, and the calcium release-activated calcium channel Orai (which does not have a P-loop) has six Glu residues on a helix with a diagonal oxygen distance of 8 Å (in the solved open-state structure)^35^.

We devised a protocol to sample selectivity filter arrangements with different geometries. First, we oriented the backbone (N-Cα-C atoms) coordinates of the Orai channel Glu residues (E106) that form the selectivity filter in the x-y plane with the z axis as the symmetry axis, discarding all but one of the symmetric mates. Next, we sampled random translations of the coordinates of this remaining residue within the x-y plane. We refer to the distance (R_s_) between the Cα atom of this residue and the central Ca^2+^ ion as the radius of the selectivity filter (Fig. 1a). We then placed a second residue with unspecified amino acid identity at a displacement (h) below the selectivity filter residue along the z axis at a distance of (R_B_) to the z axis, to define the radius of the pore exit (Fig. 1a). Finally, we applied four- or sixfold symmetry (C4 or C6) to generate new pore geometries (Fig. 1b). The initialization with the Orai E106 coordinates serves only to ensure that the Glu or Asp is roughly oriented towards the center of the pore, as the subsequent x-y translation largely eliminates dependence on these starting coordinates. We focused on C4 and C6 symmetries, as these are the most common symmetries of Ca^2+^ coordination states^25^. We selected eight filter geometries with R_s_ values evenly sampled between 5 Å and 8.5 Å for the C4 case, and 14 geometries with R_s_ values between 5 Å and 11.5 Å for the C6 case for full protein scaffold construction (discussed in the next section).

## Scaffold generation and sequence design

Using the RoseTTAFold-based diffusion method RFdiffusion^36^, the selectivity filter and exit pore defining residues were connected using helices to form the lining of the homooligomeric pore, and these helices were then extended into multi-secondary structure interacting subunits with L residues in each monomer (Fig. 1c-f). As the topology of the subunits is completely unspecified other than the pore lining helix, the diffusion process generates a wide diversity of solutions. Together, the selectivity filter generation and RFdiffusion scaffolding calculations generate multipass transmembrane protein backbones with central channels and different filter geometries. Sequences were designed using the deep learning-based protein sequence design method ProteinMPNN^37^ with position-specific amino acid constraints: the selectivity filter residues were fixed at Glu or Asp, the lipid-facing surface residues were constrained to be hydrophobic, and the remaining pore-lining residues were disfavored to be charged to avoid interference with ion permeation through the pore (see Methods). A diverse set of designed channels with varying selectivity filter geometries (the tetramers are denoted CalC4, and the hexamers CalC6) were predicted by AlphaFold2^38,39^ to assemble into structures that closely resembled the design models (predicted templating modeling (pTM) score greater than 0.8 and Cα root-mean-square-deviation (RMSD) less than 1 Å, Fig. 1g-h and Extended Data Fig. 1). Designs with many hydrophobic residues extending beyond the defined lipid-embedded regions were discarded, and 23 designs in the CalC4 series, 24 designs in the CalC6 series, and 24 designs in the CalC6_H featuring expanded selectivity filter sizes (In the H (hollow) series, the diagonal Cα distances range from 18.5 to 23.5 Å compared to 14 to 18 Å in the CalC6 series) were selected for experimental characterization.

## Characterizing designed Ca^2+^ channels

To identify functional channels, we used a cell-based flux assay with Fura-2 AM as the ion-responsive dye and Ba^2+^ as the surrogate ion for Ca^2+^ to screen for designs showing divalent ion permeability in cells. The genes encoding the designs were inserted into the LentiGuide-BC vector^40^ and were expressed in HEK293T cells by lentiviral transduction to generate a relatively homogeneous expression level of the designed proteins (see Methods). 5 of 23, 6 of 24, and 9 of 24 designs in the CalC4, CalC6 and CalC6_H series, respectively, led to increased Fura-2 fluorescence in response to 2 mM Ba^2+^ compared to the baseline when expressed in cells (Extended Data Fig. 2), consistent with channel formation.

The designs which increased Ba^2+^ flux were expressed in *Escherichia coli* (E. coli), purified by Ni-NTA affinity chromatography from the membrane fraction, and analyzed by size exclusion chromatography (SEC) and negative-stain electron microscopy (ns-EM). 3, 2 and 4 designs in CalC4, CalC6, and CalC6_H series, respectively, eluted as expected for the target oligomeric states in SEC, and formed pore-containing particles on ns-EM grids (Extended Data Fig. 3). CalC4_24, CalC6_3 and CalC6_H4 yielded the most homogeneous particles in each design group when imaged by electron microscopy (Fig. 2). CalC4_24 contains a constriction of ~1 Å in radius lined by four Glu residues, CalC6_3, a constriction of ~3 Å lined by six Glu residues, and in CalC6_H4, six Glu are placed at ~7 Å (Fig. 2a-c; pore radius calculated using MOLEonline^41^). For these designs, the peak elution fraction on SEC (Fig. 2d, ~ 12.5 mL) yielded homogeneous protein particles of ~8 nm in diameter evident in ns-EM micrographs and 2D class averages (Fig. 2e), consistent with the design models. Circular dichroism (CD) spectroscopy showed characteristic alpha-helix spectra for all three designs; CD melting experiments suggested these designs did not unfold at 95 °C (Extended Data Fig. 4). Structural similarity analysis using Foldseek-Multimer^42,43^ indicated that all the three designs are structurally distinct from the structures in the PDB and BFMD databases (Extended Data Fig. 5 and Supplementary Table 2-4). AlphaFold3^44^ predictions showed Ca^2+^ ions at the highly negatively-charged designed sites^45,46^ in CalC4_24 and CalC6_3, albeit with relatively low confidence levels (pLDDT (predicted local distance difference test) less than 80, Extended Data Fig. 6).

Next we sought to investigate the conductances of the designed channels for Ca^2+^ by whole-cell patch-clamp recordings on insect *Trichoplusia ni* cells (Hi5) expressing the designs. An experimental challenge is to subtract the background currents (from the leak between the patch pipette and the cell membrane, and from all other channels present in these cells) from the ionic currents for the designed (and constitutively open) channels. We used N-methyl-D-glucamine (NMDG^+^) and methanesulfonate (MeSO_3_^−^) to replace inorganic ions in both extracellular and intracellular solutions (Fig. 3a), bathed the cells in 0.02 mM [Ca^2+^] solution to obtain the baseline currents, and then increased the Ca^2+^ concentration 500 fold to 10 mM [Ca^2+^] solution to preferentially record Ca^2+^ currents (see Methods). With this experimental setting, inward currents at −100 mV were observed to increase for both CalC4_24 and CalC6_3 in 10 mM [Ca^2+^] bath solution. This increase of inward currents was reversible and was diminished when the bath solution was changed back to 0.02 mM [Ca^2+^] solution (Fig. 3b-c). The I-V relations of both channels obtained from both a −100 mV to +100 mV (with a holding potential of 0 mV) voltage step protocol (Fig. 3d-h) and a voltage ramp protocol (Fig. 3i-k) exhibited sharp inward rectifications, as expected based on our experimental design (Ca^2+^ is only present extracellularly and hence can only give rise to inward currents). A current density of approximately 4 pA pF^−1^ at −100 mV in the 10 mM [Ca^2+^] solution was consistently present in cells infected with control baculovirus that did not encode the designs (made from an empty pFastBac-Dual vector), even with 4 mM MgATP included in the patch pipette (Fig. 3l-m and Extend Data Fig. 7). This current was likely the result of a promiscuous endogenous Ca^2+^-permeable channel(s) in Hi5 cells, as it exhibited inward-rectification as well. The current densities in cells expressing CalC4_24 and CalC6_3 were determined to be 7.5 pA pF^−1^ and 9.1 pA pF^−1^, respectively, both significantly larger than the background current (Fig. 3l-m). We did not proceed with testing the CalC6_H4 design by whole-cell patch-clamp experiments due to the possibility of additional ions such as NMDG^+^ or MeSO3^−^ also permeating through the pore, making separation of leak currents more difficult.

To examine the specificity of the channels for different cations, the 10 mM Ca^2+^ in the extracellular solution was substituted with 40 mM Na^+^ ,10 mM Sr^2+^ or 10 mM Mg^2+^ (the Na^+^ concentration was four times that of the divalent ions as the current scales with the square of the ion valence; see Supplementary information). The relative permeabilities of ions through ion channels are often calculated from the reversal potential using the Goldman-Hodgkin-Katz (GHK) voltage equation when two ions with known concentrations are put on the extracellular and intracellular sides, respectively^47,48^. However, in our case this is complicated by the concentration gradient-driven diffusion through the open channels, which results in variation in the ion concentrations, and the contribution of endogenous channels to the reversal potential is difficult to disentangle. We instead compared conductances for each cation based on the inward current at −100 mV using the GHK flux equation (not the voltage equation, see Methods). Both CalC4_24 and CalC6_3 showed ~5 fold larger current in 10 mM [Ca^2+^] than in 40 mM [Na^+^] solution at −100 mV (Fig. 4a-d, Extended Data Fig. 7). The relative conductances of the divalent cations for both designs followed the order Ca^2+^ > Sr^2+^ > Mg^2+^ (Fig. 4e-h), as observed for several native Ca^2+^ channels (Ca_v_, TRPV6, CRAC)^30,49–54^. Likewise, both channels were blocked by 10 μM La^3+^, which blocks native Ca^2+^ channels (Fig 4i-l).

Both CalC4_24 and CalC6_3 exhibit electrophysiological properties expected for Ca^2+^ channels: inward rectification in presence of extracellular Ca^2+^, higher relative conductances to Ca^2+^ than to other cations, and blocking by lanthanum. Thus, our approach of arranging Glu/Asp carboxylate residues around a central pore generates Ca^2+^ dependent channels as intended. Although the selectivity filter of CalC6_3 (6.4 Å) is much wider than that in CalC4_24 (2.5 Å, both distances represent that between diagonally opposed oxygen atoms), the electric field strength near the filter is probably stronger in CalC6_3 due to the presence of two extra Glu residues, which likely facilitates recruitment of Ca^2+^ ions. The adjacent Glu residues are predicted by AlphaFold3 to interact and partially dehydrate the Ca^2+^ ion (Extended Data Fig. 6a-b), as in the selectivity filter configuration observed in the crystal structure of the Ca_v_ab channel in complex with dihydropyridine compounds^32^. In our experiments 4 mM MgATP (free [Mg^2+^] estimated to be approximately 400 μM in the presence of 20 mM EGTA^55^) was included in the patch pipette to inhibit a large endogenous non-selective cation current, and this could also reduce the currents observed for Na^+^ and other divalent ions (AlphaFold3 also predicts some binding of Mg^2+^ to the selectivity filter; Extended Data Fig. 6c-d), consistent with observations that Mg^2+^ can bind to selectivity filters but not readily permeate in some native Ca^2+^ channels^6,54,56^. The relative conductances order Ca^2+^ > Sr^2+^ > Na^+^ of the designs likely reflects a preferential binding of ions at the selectivity filter with a high field strength (HFS).

## Role of selectivity filter residues

To ascertain the accuracy of design of the selectivity filter geometry and overall channel architecture, we sought to determine the structure of CalC6_3 using cryo-electron microscopy (cryo-EM). CalC6_3 in detergent particles appeared homogeneous after being frozen on the grid (Extended Data Fig. 8b) and 4,644 movies were collected; however, top views of the particles could not readily be separated from side views during 2D classification due to the spherical shape of the detergent-bound membrane protein, a well-known challenge for structural determination of relatively small membrane proteins without obvious soluble domains^57^. To facilitate structural determination, we applied a fusion protein strategy: we connected structurally-validated designed helical repeat (DHR) proteins^58^ to the C-terminus of CalC6_3 via straight helices, with linker helices generated using RFdiffusion and sequences designed using ProteinMPNN (see Methods, Extended Data Fig. 8e). Among the six tested channel-DHR fusion proteins, one showed quite homogeneous particles on the ns-EM grid.

We proceeded with cryo-EM data collection with this construct, and obtained a three-dimensional reconstruction to a global resolution of 3.75 Å with C6 symmetry (Fig. 5a,e and Extended Data Table 1), and were able to build a structure model based on the density map (Fig. 5b and f). The density for the DHR extension domains were not well resolved possibly due to structural flexibility. We were also able to identify some minor heptamer and octamer species from the 2D classification, and obtained 3D reconstructions for the heptamer to an overall resolution of 4.62 Å with C7 symmetry. (Extended Data Fig. 9 and Extended Data Table 1). The existence of multimeric proteins in an additional oligomeric state has been observed in naturally-existing proteins, for example calcium homeostasis modulator (CALHM) family members^59,60^.

In the cryo-EM structure of the hexamer, the density for the Glu-ring selectivity filter density and the His-ring below the filter was clearly evident when contoured at and below 4 σ level (Fig. 5c and g), suggesting pre-organization of the Glu-ring by the His-ring via potential hydrogen bond interactions. Although the sidechains were not well resolved given the resolution of the cryoEM map, the geometry of the selectivity filter, defined by the Cα atoms of the Glu residues, was almost exactly as designed (backbone RMSDs of 1.04 Å and 1.26 Å for monomer and complex alignment, respectively; Fig. 5d and h).

To directly test the function of the glutamate selectivity residues of CalC6_3, we mutated them to leucine and measured the Ca^2+^ conductance. The predicted structure of the E101L mutant was almost identical to that of CalC6_3 (Fig. 5i). The purified E101L protein assembled into homogenous pore-containing particles by ns-EM indistinguishable from the wild-type (E101) pore (Fig. 5j), and in Hi5 cells the E101L mutant expressed at the same level as the wild-type design (Fig. 5k). Electrophysiology measurements showed that the leucine substitutions reduced the Ca^2+^ conductance of the CalC6_3 channel to that of the negative control (~ 4 pA pF^−1^ at –100 mV, Fig. 5l). As an independent test, we used a luciferase reporter under the control of a Ca^2+^-responsive transcription factor NFAT (nuclear factor of activated T cells) response element (NFAT-RE); calcium treatment (culturing in the DMEM medium) of cells expressing the wild type design resulted in significantly more luminescence than did treatment of the E101L mutant (Extended Data Fig. 10). Since the overall structure of the pore is not affected, and the size of the orifice formed by the Leu residues is nearly identical to that formed by the Glu residues, this high sensitivity to negative charge to nonpolar substitution strongly suggests that ions flow through the central pore as designed, and that direct interactions between selectivity filter residues and the permeant ions determine ion selectivity and flux as in naturally occurring channels.

## Discussion

De novo design of ion channels is in principle a powerful approach to study ion channel biophysics and to develop new biological tools, but the ability to tune ion selectivity has been limited by lack of an approach to install selectivity filters with specified composition and geometry into designed channels. We demonstrate that using generative RFdiffusion symmetric motif scaffolding, protein topologies can be built from scratch to optimally scaffold selectivity filters with pre-defined geometries. In contrast, previously design efforts sought to change conductances by altering the number of alpha helices or beta strands surrounding the pore, rather than building up from defined selectivity filter geometries and compositions. Ca^2+^ selective channels were not obtained using these previous approaches, which is expected given the challenge of selectively recognizing Ca^2+^ ions while maintaining a rapid Ca^2+^ ion flow. In contrast, the designed CalC4_24 and CalC6_3 channels have precisely designed selectivity filter geometries, and have higher conductances for Ca^2+^ than for other cations. The high degree of agreement between the cryo-EM structure and the design model of CalC6_3 demonstrates the accuracy of the design method. That calcium selectivity can be achieved using rings of carboxylate containing residues in our designs is a proof by direct construction that interactions between ions and multiple sidechain carboxylate groups can impart selectivity, and the considerable diversity in detailed selectivity filter geometry in our designs and native channels suggests further that the detailed geometry of these interactions can be quite varied. Our approach enables the exploration of diverse selectivity filter geometries and chemical compositions for optimal ion channel conductance and selectivity, which are difficult to probe through classical native ion channel mutagenesis experiments: for example, it will be interesting to explore how increasing selectivity filter complexity (such as breaking symmetry), and incorporating additional filter layers, impacts channel selectivity. Building up channels around defined selectivity filter geometries should both enable rigorous testing of our understanding of the determinants of native ion channel selectivity decoupled from complex channel activation and deactivation processes (native channels have binding sites for signaling molecules such as calmodulin and phosphatidylinositol phosphates that modulate channel function), and the construction of a wide array of new channels with selectivities that go beyond those observed in nature. Because of their simplicity, modularity and lack of sequence homology to naturally occurring proteins, our Ca^2+^ channels provide attractive starting points for new biorthogonal tools for modulating cellular calcium flux.

## Methods

### 1. Computational design methods

#### 1.1. Placement of pore helices

The pore helices can be generated in three steps: 1) placement of Ca^2+^-coordinating residues as the selectivity filter, 2) placement of pore exit residues, and 3) generation of protein backbones to hold these pore-defining residues.

In theory any structural element containing carboxylates and Ca^2+^ ions can be used to define the filter. We extracted the six Glu residues from the open-state structure of Orai channel as the template Ca^2+^-coordinating motif to start from. The coordinates of the motif were changed simultaneously such that the motif was placed in the x-y plane with the Ca^2+^ ion on the z axis using PyMOL. Then five copies of the Glu/Asp residues were deleted, and the x and y coordinates of the remaining Glu/Asp residue were sampled using PyRosetta through rigid-body moves to generate ion-residue pairs with different distances. These ion-residue pairs were symmetrized using the SetupForSymmetry Mover in RosettaScripts with applied C4 or C6 symmetry to generate selectivity filters with different geometric parameters.

The pore exit residues were generated by positioning a set copy of the Ca^2+^-coordinating residues at specific distances (h) along the z axis away from the original x-y plane. This positioning was applied as rigid body transformation in Pyrosetta. The z-distance between the two layers of residues defined the length of the pore. The ideal z-distance was determined to be 43.2 Å in our case mainly based on three considerations: (1) the vertical distance between residue i and i+8 that have the same sidechain orientation (i.e., two consecutive helical turns) is 10.8 Å, (2) the membrane thickness in eukaryotic cells is usually greater than 30 Å^61,62^, and (3) we want the pore helices to extend beyond the lipid bilayer, which can reduce the overall hydrophobicity of these helices, and in turn reduce the trend of protein aggregation and non-specific interhelical packing interactions driven by large patches of hydrophobic residues. So the pore length is determined to be 4 × 10.8 Å = 43.2 Å. At this stage the specific amino acid identities of the pore exit residues are inconsequential, as the sequence will be redesigned by ProteinMPNN at a later sequence design step. The x and y coordinates of these pore exit residues were sampled using the rigid body transformation function of Pyrosetta to generate pores with different geometries.

To form the lining of the pore, RFdiffusion was used to symmetrically generate helices that connected the selectivity filter and pore exit residues. The number of the helical linker residues was sampled in order to generate straight alpha helices as the optimal secondary structure. Typically 25–29 residues were sufficient to position the pore entrance and exit residues on a straight helix, depending on the tilt angle of the pore helix relative to the z axis.

#### 1.2. Generation of supporting protein backbones

Once the pore helices were obtained, RFdiffusion was subsequently used to symmetrically generate protein backbones for extending the pore helices into homo-oligomeric subunits with L residues in each monomer. The overall length of the monomer (L) largely depends on the number of times it passes through the membrane and relative distances between neighboring chains. In particular, the number of residues before (at N-terminal of) and after (at C-terminal of) the pore helices were sampled to ensure adequate buttressing of protein backbones within each monomer and extensive interface areas between neighboring subunits. In general, we found that 30–40 residues were sufficient to form a straight single-pass helix, and could be used as the repeating unit to estimate a rough range of the residue numbers needed to generate. The backbone outputs were examined by eye, and those with well-buttressed helices and open pores were selected for the subsequent protein sequence design.

We separated the processes of the generation of pore helices and the scaffolding of pore helices although both steps were done by RFdiffusion. This appeared to be more effective in guiding RFdiffusion to make more compact ion channel-like helical structures than only scaffolding the selectivity filter and pore exit residues. In the latter case, the output backbones mostly contained fiber-like helical coiled-coil assemblies extending far away from the selectivity filter.

#### 1.3. Fine-tuning RFDiffusion on transmembrane proteins

When the diameter of the pore exit is large (> 20 Å between diagonal Ca atoms), the published RFdiffusion model^36^ tends to generate protein fragments inside the pore, thus occluding the ion permeation pathway. This is likely due to the globular nature of soluble proteins that constituted the majority of the training dataset for RFdiffusion. To overcome this, we fine-tuned RFdiffusion on a dataset containing 6,392 transmembrane proteins from the OPM (https://opm.phar.umich.edu/) database comprising all beta-barrel and alpha-helical proteins as of April 2023 to generate backbones with higher chances of maintaining a pore. Specifically, we fine-tuned the version of RFdiffusion trained with (partial) secondary structure and block-adjacency information provided, and continued to provide such information during fine-tuning, such that at design time one can specify the desired protein topology. We fine-tuned RFdiffusion in precisely the manner described in Watson et al., 2022, and trained for 100 epochs on this transmembrane dataset.

#### 1.4. Sequence design on protein backbones

The selected protein backbones of the channels were sequence-designed with ProteinMPNN. The --tied_positions argument was used to restrict sequences to be identical on each monomer. The residues forming the selectivity filter were kept undesigned using the --fixed_positions argument. Position-specific amino acid constraints were applied using the --omit_AAs argument. The pore-lining residues were selected based on the distance between the Cα atom of the residue and the central z axis (the square root of the sum of the squares of x and y coordinates of that CA atom in the PDB file). Charged and bulky aromatic amino acids (tyrosine and tryptophan) were excluded from the selected pore residues to prevent interference with ion permeation through the pore, particularly through sites that might form narrow constrictions. Residues that were defined as being embedded in the lipid bilayer were selected based on their relative vertical (z) distances to the selectivity filter. The selectivity filter residues were arbitrarily designated as the initial point of the lipid bilayer on the extracellular side. Residues that are within a certain range on the z-distance (e.g., 30 Å) were all defined to be embedded in the lipid bilayer. The lipid-facing residues were selected combinatorially by (1) the Rosetta LayerSelector using the sidechain neighbor algorithm, (2) not being among the pore-lining residues, and (3) being among the lipid-embedded residues. Tyrosine (Tyr) and tryptophan (Trp) residues were placed at the first and final residues of the surface residues on a consecutive helix, respectively, interfacing the aqueous phase and lipid phase.

It is optional to use the ProteinMPNN model trained with extra input per residues specifying buried and interface residues^63^ using the argument --model_type “per_residue_label_membrane_mpnn”. In this case the lipid-facing residues were parsed into ProteinMPNN using the --transmembrane_buried argument. Other constraints were applied in the same way as described above.

#### 1.5. Structure prediction on designed sequences

AlphaFold2 was primarily used to judge if the designed sequences would fold and assemble into homo-oligomers as designed. Model 4 seemed to be most predictive of the designed alpha-helical transmembrane proteins and was the only model used. Designs were first filtered by confidence scores (typically pTM > 0.8 and mean PAE scores < 10 for all residue pairs corresponding to different chains) and then manually inspected to exclude designs with clusters of hydrophobic residues outside the defined lipid-embedding regions.

For estimating ion binding at the selectivity filters, AlphaFold3 was used to predict the formation of protein-metal complexes. Note that there could be clusters of negatively charged residues on the surface of the designed proteins forming potential ion binding sites. Therefore, multiple copies of ions were often included as the input rather than a single ion.

#### 1.6. Resampling and optimization of protein backbones

In some cases, very few designed sequences were predicted to fold with high confidences for some specific protein backbones with very tilted pore geometries. We hypothesized that the low in silico success rate was due to non-ideality of the protein backbones, which resulted in a low “designability” by ProteinMPNN. To refine the protein backbones, we applied partial noising and denoising processes using RFdiffusion partial diffusion to sample for more reasonable structures around the original backbone. This resampling process was not applied on the pore helices to maintain the pore geometry. The newly generated backbones were then subjected to another round of backbone selection, sequence design, and structural prediction as described above.

#### 1.7. Connecting DHR to designed oligomeric channel for Cryo-EM analysis

To connect DHR proteins as the soluble domain to CalC6_3 for structural determination, the main objective is to avoid potential steric clashes between the subunits after addition of multiple copies of DHR proteins. This was achieved by precisely adjusting the relative position of the DHR proteins to CalC6_3, and this positioning operation was primarily done in PyMOL. The N-termini of experimentally-validated DHR proteins with different twisted geometries were aligned to the C-terminus of the CalC6–3 monomer and were oriented to face away from the pore. Then the DHRs were moved in the direction parallel to the aligned helix away from CalC6_3 to a point where the DHRs were unlikely to clash with the neighboring subunits. RFdiffusion was subsequently used to generate straight helices to fuse DHRs to the CalC6_3 monomer. The linked structure was symmetrized using the Rosetta SetupForSymmetry Mover by generating five extra copies. The residues that might potentially interact with neighboring subunits were manually selected and redesigned by ProteinMPNN as homo-hexamer. The sequence of the redesigned monomer was predicted by AlphaFold2 (the whole complex was too large for AlphaFold2 to predict). Designs with lowest Cα RMSDs to the CalC6_3 model in regard to the channel region were selected for experimental characterization.

### 2. Experimental methods

#### 2.1 Construction of synthetic genes

The amino acid sequences of the designs were reverse translated to DNA sequences using DNAworks (Hoover, D. and Lubkowski, J., 2002, https://github.com/davidhoover/DNAWorks). Synthetic genes encoding the designs were ordered from Genscript Inc. (Piscataway, N.J., USA) or Integrated DNA Technologies, Inc. (IDT, Coralville, Iowa, USA). The genes encoding the designs for protein expression and purification were cloned into pET29b+ vector between NdeI and XhoI sites with a C-terminal hexahistidine tag. The genes encoding the designs for expression in HEK293T cells were cloned into the LentiGuide-BC-EF1a vector between XbaI and EcoRI sites with a C-terminal FLAG tag (DYKDDDDK). The genes encoding the designs for expression in insect cells were cloned into the MCS-1 of pFastBac-Dual vector between BamHI and XbaI sites with a C-terminal hexahistidine tag.

#### 2.2. Expression of designed channels in HEK cells

HEK293T cells (from ATCC) were maintained at 37 °C with 5% CO_2_ and cultured in Dulbecco's Modified Eagle's Medium (DMEM, Gibco) supplemented with 10% FetalClone II serum (FC-II, Cytiva HyClone) and 1% penicillin-streptomycin (Gibco). To favor a more homogeneous expression level for more consistent flux assay results, we expressed the designed channels in HEK293T cells by lentivirus transduction based on a published protocol with some minor modifications^40^.

To prepare the lentivirus, cells were transfected with the design plasmid, the envelope plasmid pMD2.G (Addgene #12259) and the packaging plasmid psPAX2 (Addgene #12260) at a 3:4:5 mass ratio using Lipofectamine 3000 (Thermo Fisher L300015). Media was exchanged after 4 hours. At 24 hours post-transfection, caffeine was supplemented to a final concentration of 2 mM to generate virus with higher titer. The virus-containing supernatant was harvested 72 hours after transfection and filtered through 0.45 μm surfactant-free cellulose acetate (SFCA) filters (Corning 431220). The prepared viruses were used immediately or stored at −80 °C.

For lentiviral transduction, cells were dissociated from the culture flask using 0.05% trypsin-EDTA (Gibco) and resuspended in DMEM-10% FC-II medium supplemented with polybrene (8 μg/mL, Santa Cruz Biotechnology) at a cell density of 1,000,000 cells/mL. Viral supernatant was added to this cell suspension (typically 200 μL viral supernatant was used to transduce 1 mL of the above cell suspension). The cells-virus mixture was centrifuged at 1000 g for 2 hours at 33 °C. Media was exchanged 24 hours after infection.

#### 2.3. Flux assay of designed channels in HEK cells

At 48 hours post-infection, transduced cells were seeded into glass-bottom 96-well plates (Cellvis P96–1.5H-N) pretreated with 0.05% poly-D-lysine hydrobromide (Sigma, dissolved in sterile-filtered Milli-Q water) at a density of 60,000 cells per well. A Ca^2+^-free HEPES-buffered solution (20 mM HEPES pH 7.4, 150 mM NaCl, 5 mM KCl, 2 mM MgCl_2_, 10 mM glucose, osmolarity adjusted to 330 mmol/kg using sucrose) was used as the assay buffer. To prepare the dye-loading solution, a vial of Fura-2 AM dye (Invitrogen F1221, 50 μg) was thawed, resuspended in 10 μL DMSO, and added to the assay buffer supplemented with Powerload (Invitrogen P10020) and probenecid (Invitrogen P36400) to a final dye concentration of 5 μM. Cell culture media was replaced with this dye-loading solution and incubated for 1 hour at room temperature. After the dye loading step, the cells were washed with the assay buffer twice, incubated in the assay buffer, and measured for fluorescence at 510 nm with excitations at 340 nm and 380 nm (F340/F380) using the BioTek Synergy Neo2 plate reader. The measurement was performed for 15 minutes to obtain the baseline fluorescence. Subsequently, Ba^2+^ was added to the cells to a final concentration of 2 mM and the fluorescence was measured for 15 minutes to obtain the change of fluorescence ratio over time. Data were exported as Excel tables, processed and plotted using Python.

#### 2.4. Protein production in E.coli

The designs in pET29b+ vector were transformed into E. coli expression strain BL21(DE3*) (New England Biolabs, MA, USA). The transformed cells were grown in 50 mL Terrific Broth II (TB-II, MP Biomedicals) medium with a final concentration of 50 μg/ml kanamycin in 250 mL baffled flasks overnight. The 50 mL cultures were inoculated into 500 mL TB-II medium in 2 L baffled flasks and incubated at 37 °C. After 3 hours, isopropyl β-D-1-thiogalactopyranoside (IPTG) was added to a final concentration of 0.5 mM. The cultures were incubated at 18 °C following the addition of IPTG and were harvested after 3 hours by centrifuging at 4000 g for 10 minutes at 12 °C. The cell pellets can be stored at −80 °C.

#### 2.5. Buffer recipe for protein purification

Lysis buffer: 20 mM Tris-HCl, 150 mM NaCl, pH 8.0, supplemented with Pierce Protease Inhibitor Tablets (Thermo Scientific A32963, 1 tablet per 100 mL solution)

Solubilization buffer: 20 mM Tris-HCl, 150 mM NaCl, pH 8.0, supplemented with 2% w/v n-Decyl-β-D-Maltopyranoside (DM, Anatrace D322)

Wash buffer 1: 50 mM Tris-HCl, 300 mM NaCl, 30 mM imidazole, pH 8.0, supplemented with 0.06% w/v glyco-diosgenin (GDN, Anatrace GDN101)

Wash buffer 2: 50 mM Tris-HCl, 1 M NaCl, 30 mM imidazole, pH 8.0, 0.06% GDN

Wash buffer 3: 50 mM Tris-HCl, 300 mM NaCl, 60 mM imidazole, pH 8.0, 0.06% GDN

Elution buffer: 50 mM Tris-HCl, 300 mM NaCl, 500 mM imidazole, pH 8.0, 0.06% GDN

SEC buffer 1: 20 mM Tris-HCl, 150 mM NaCl, pH 8.0, 0.06% GDN

SEC buffer 2: 20 mM Tris-HCl, 150 mM NaCl, pH 8.0, 0.006% GDN

#### 2.6. Purification of designed calcium channels

The cell pellets were resuspended in 30 mL lysis buffer. The suspension was placed on ice and lysed by sonication (QSonica Sonicators, CT, USA) for 4 minutes (15 seconds on/15 seconds off, 8 minutes total run time) at 65% power with ¾’’ Dia replaceable tips. The lysates were centrifuged at 10000 g for 15 min to remove the cell debris. The supernatant was collected and ultracentrifuged at 170,000 g for 1 hour at 12 °C using Beckman Optima XE-90 Ultracentrifuge to pellet down the membrane fraction. 8 mL solubilization buffer was added to the pellet and incubated at 4 °C overnight on a rocker. The homogenate was centrifuged at 30,000 g for 30 minutes to pellet down the materials that could not be solubilized by DM. The supernatant was collected and applied to chromatography columns (BIO-RAD Econo-Pac gravity flow columns) containing Ni-NTA resin (Qiagen, MA, USA). The resin was washed with 5x column volume (CV) of wash buffer 1, 5x CV of wash buffer 2 and 3x CV of wash buffer 3. The proteins were eluted with 4x CV of elution buffer, concentrated in 100 kDa molecular weight cutoff spin concentrator (Millipore), and further purified by size exclusion chromatography (SEC) in SEC buffer 1. Normally a Superdex 200 Increase 10/300 GL column was used. For designed proteins connected with DHR, a Superose 6 increase 10/300 column was used. Both types of columns were from Cytiva, MA, USA. The target elution volume containing the desired oligomeric states of the designs was compared with validated de novo designed transmembrane proteins with similar molecular weight (for example, TMH4C4, which approximated a molecular weight of around 100 kDa, and TMHC6, which approximated a molecular weight of around 60 kDa).

In many cases, there seemed to be an evident aggregation peak when samples were applied on the SEC column after Ni-NTA purification (Fig. S2). This is a well-known issue for the purification of membrane proteins, whereby the behavior of membrane proteins in vitro is largely dependent on the detergent or lipids utilized. We took a similar purification strategy as for purifying mammalian voltage-gated sodium channels^64–68^: we took the latter portion of the elution peak containing the proteins that were estimated to be in the desired oligomeric states, and subjected this portion to the a second SEC run in a buffer with ten 10 times less detergent (SEC buffer 2). The second SEC yielded a more distinct peak corresponding to the protein in the desired oligomeric states, which enabled us to separate the species of interest. 0.006 % GDN was used in the final buffer, as this consistently gave the optimal EM results.

#### 2.7. Circular dichroism (CD) measurements

CD spectra were measured on J-1500 Circular Dichroism Spectrophotometer (Jasco). Proteins were prepared at ~0.2 mg/mL in SEC buffer 2. Wavelength-scan spectra were recorded from 190 nm to 260 nm (0.1 nm increments). Temperature melting experiments were conducted from 25 °C to 95 °C (heating rate 1 °C/min), with wavelength-scan spectra recorded at every 10 °C increment. The full spectra were measured again after the samples were cooled down to 25 °C.

#### 2.8. Negative-stain electron microscopy

Proteins were prepared at ~0.02 mg/mL in SEC buffer 2 for ns-EM. 6 μL of the protein sample was applied on glow discharged, carbon-coated 400-mesh copper grids (Electron Microscopy Sciences). The samples were allowed to adhere to the grids for 1 min before being wicked away. Then each grid was stained with 3 μL of 2% uranyl formate for four times, each time 20 seconds. The grids were air-dried and were imaged on a FEI Talos L120C TEM (FEI Thermo Scientific, Hillsboro, OR) equipped with a Ceta 4K CCD camera at a magnification of 73,000x at 120 kV. Micrographs collection was automated using the EPU software (FEI Thermo Scientific). Collected datasets were imported into CryoSPARC software (v4.6.0) for 2D class averaging. Micrographs were motion-corrected and CTFs were estimated using Patch CTF estimation function. For each dataset, ~200 particles were selected using the manual particle picker function to generate initial 2D class averages using the 2D classification program in CryoSPARC. The 2D class averages were used as templates for automated particle picking using the template particle picking function after which 50 classes of 2D class averaging were generated for analysis.

#### 2.9. Cryo-EM Sample preparation

For CalC6_3, 3 μL of protein at ~ 1 mg/mL in SEC buffer 2 (20 mM Tris-HCl pH 8, 150 mM NaCl, 0.006% GDN) was applied to glow-discharged (25 seconds at 15 mA) 3 nm lacey carbon Cu 400 mesh grids (Electron Microscopy Sciences).

For CalC6_3 with DHR extensions, protein was prepared at ~1 mg/mL in SEC buffer 2 (20 mM Tris-HCl pH 8, 150 mM NaCl, 0.006% GDN). 2 μL of protein sample was applied to glow-discharged (25 seconds at 15 mA) C-flat R 2.0/2.0 300 mesh holey carbon grids (Electron Microscopy Sciences), and 3 μL of protein sample was applied to glow-discharged (16 seconds at 5 mA) Quantifoil 2 nm thin carbon R 2.0/2.0 300 mesh grids.

Vitrification was performed using a Mark IV Vitrobot at 22 °C and 100% humidity for all grids. Blotting was done before immediately plunge-frozen into liquid ethane. A blot time of 0.5 second, a blot force of 0 and a wait time of 7.5 second were used for lacey carbon grids and Quantifoil thin carbon grids. A blot time of 6.5 second, a blot force of 0, and a wait time of 7.5 second were used for holey carbon grids. The grids were then clipped and stored in liquid nitrogen for collection on the Titan Krios.

#### 2.10. Cryo-EM data collection

Samples were collected automatically using SerialEM^69^, used to control a ThermoFisher Titan Krios 300 kV microscope equipped with a K3 Summit direct electron detector^70^ and BioQuantum Gif energy filter. For CalC6_3 with DHR extensions (thin carbon and holey carbon grids) the microscope was operated in counting mode. For CalC6_3 (lacey carbon grids) super resolution mode was used. Data were collected with random defocus ranges spanning between −0.8 and −1.8 μm using beam-image shift five shots per hole and nine holes per stage move were collected for CalC6_3 with DHR extensions (thin and holey carbon grids) and 27 shots per stage movement (3×9) for CalC6_3. Altogether 6,893, 7,376, and 4,644 movies with pixel sizes of 0.843, 0.843, and 0.4135 and doses of 47.32, 47.32, and 60 e-/Å^2^ were recorded for CalC6_3 with DHR extensions on thin carbon grid, holey carbon grid, and CalC6_3 on a lacey carbon grid, respectively.

#### 2.11. Cryo-EM data processing

All data processing was carried out in CryoSPARC v4.4^71^. For all movies Patch Motion then Patch CTF data processing was carried out as an initial processing step. Custom settings are mentioned for refinement jobs in the protocol, otherwise default settings were used.

For CalC6_3: Curate Exposures was used to remove 1,078 micrographs, leaving 3,566 good micrographs. Blob Picking was used with a minimum particle diameter of 80 Å and a maximum particle diameter of 120 Å. An extraction box size of 300 pixels, and 680 pixels fourier cropped to 340 pixels was used. Following an Inspect Picks job 1,311,993, and 1,094,418 particles were extracted for 300 and 680 pixel sizes, respectively. Extensive 2D classification was performed on the 300 pixel size particles, however particles could not be effectively classified. The larger extraction box size of 680 yielded some low resolution top views showing the pore (Extended Data Fig. 8a-d). Further 2D classification and possible 3D refinement was abandoned in favor of the CalC6_3 with DHR extensions.

For CalC6_3 with DHR extensions: Curate Exposures was used to remove 172 and 1,217 bad micrographs, leaving 6,721 and 6,161 micrographs for thin and holey carbon grids respectively. Blob picking was used with a minimum particle diameter of 80 Å and maximum particle diameter of 160 Å. Particles were extracted at 340 pixels. Following an Inspect Picks job 8,115,000 and 7,293,711 particles were extracted for thin and holey carbon respectively. For thin carbon particles an initial round of 2D classification, 150 classes with a circular mask of 220 Å was used. From this 886,110 particles representing top and side views were further 2D classified with: 150 classes, batchsize per class of 400, 50 online-EM iterations, min over scale after first iteration set to true, with 3 final iterations using all particles. For holey carbon particles an initial round of 2D classification with: 400 classes, and number of final iterations set to 5 was used. From this 185,009 particles representing top and side views were further 2D classified: 50 classes, and 5 final iterations with all particles. Top view classification was abandoned for holey carbon as no secondary structure could be resolved. From the holey carbon 142,660 particles were identified as side views by the visible DHR extensions. (Extended Data Fig. 8e-i)

For the hexamer structure only the thin carbon data was used. 115,880 particles of top/tilted views were selected from the previous 2D classification job. 110,256 particles with side views were further 2D classified separately using: A maximum resolution and a maximum alignment resolution of 3 Å, circular mask diameter and circular mask diameter outer set to 100Å and 120Å respectively (To mask out the flexible DHR extension), a batch size per class of 400, and number of online-EM iterations set to 40 was used. Of these true side views 13,304 particles were selected where clear secondary structure was visible. These final 129,184 particles with side and top/tilted views were further classified by generating 2 *ab initio* classes with: C6 symmetry, initial lowpass resolution set to 15 Å, GSFSC split resolution set to 10 Å, and optimize per-particle defocus set to true. The best *ab initio* volume and 85,292 particles were used to generate a 4.19 Å volume using Non Uniform refinement with: C6 symmetry, initial lowpass resolution set to 15 Å, GSFSC split resolution set to 10 Å, and optimize per-particle defocus set to true. To correct for any aberrations introduced from the beam-image shift collection strategy, Exposure Group Utilities was used to split the data into 101 exposure groups, using Global CTF Refinement with settings: Fit spherical aberration set to true, fit tetrafoil set to true. The subsequent Non Uniform refinement job improved the resolution of the map to 3.79 Å using: C6 symmetry, initial lowpass resolution set to 15 Å, GSFSC split resolution set to 10 Å, and optimize per-particle defocus set to true. This 3.79 Å map was used as an input for Reference Based Motion Correction, followed by another Non Uniform Refinement which further improved the map resolution to 3.60 Å using the following custom settings: symmetry C6, number of extra final passes 5, initial lowpass resolution set to 15 Å, GSFSC split resolution set to 10 Å, and optimize per-particle defocus set to true. Using the remaining 83,766 particles and the 3.60 Å map as the input, a round of heterogeneous refinement was performed in C1 with 8 volumes. The particles from the 4 best volumes with clearly defined secondary structure were kept. 67,181 particles were used to generate the final volumes with No Uniform Refinement in C1 and C6 symmetry with: initial lowpass resolution set to 15 Å, GSFSC split resolution set to 10 Å, and optimize per-particle defocus set to true. Final resolutions were 3.75 Å and 3.98 Å for C6 and C1 maps respectively. While a slight drop in resolution was observed the map quality was much higher when visually inspected. The final C6 map used for model building was sharpened with DeepEMhancer^72^. Final maps are deposited in the EMDB under EMD-47340.

For the heptamer structure the thin and holey carbon data was used, utilizing top and tilted views from the thin carbon and side views from both the thin and holey carbon data. From the second 2D classification of the particles on the thin carbon grid, 8 classes of 89,183 particles were selected with top and tilted views that appeared to be larger than hexameric by visual inspection. 110,256 particle side views from the thin carbon grid and 142,660 particle side views from the holey carbon grid were combined and an initial 2D classification was performed with settings: maximum resolution and maximum alignment resolution of 3 Å, circular mask diameter and circular mask diameter outer of 130 Å and 150 Å respectively, batch size of 400, force max over poses/shifts set to false, number of online-EM interactions set to 40. Subsequent 2D class averages were removed that appeared hexameric, judging from the side views used in the hexamer structure. 66,778 particles were further 2D classified using: maximum resolution and maximum alignment resolution of 3 Å, circular mask diameter and circular mask diameter outer of 100 Å and 120 Å respectively, batch size of 400, force max over poses/shifts set to false, number of online-EM interactions set to 40. 46,550 particles were selected where any secondary structure was visible and an additional 2D classification was performed using: maximum resolution and maximum alignment resolution of 3 Å, circular mask diameter and circular mask diameter outer of 100 Å and 120 Å respectively, batch size of 400, force max over poses/shifts set to false, number of online-EM interactions set to 40. 20,173 particles with clear secondary structure were selected. Using the 89,183 particles of heptameric top/tilted views and the 20,173 particles from extensive 2D classification of side views additional sorting was done with an *Ab Initio* job using: 3 *ab initio* classes, C7 symmetry, maximum resolution of 6 Å, Initial resolution of 25 Å. The best *ab initio* volume was selected containing 45,077 particles, and used for a Non-Uniform Refinement job both in C7 and C1 symmetry with: number of extra final passes set to 5, initial lowpass resolution set to 15 Å, GSFSC split resolution set to 10 Å, and optimize per-particle defocus set to true. For the C7 and C1 map a resolution of 4.62 Å and 5.13 Å was achieved respectively. The final C7 map used for model building was sharpened with DeepEMhancer. Final maps are deposited in the EMDB under EMD-47356.

#### 2.12. Model Refinement

For all structures, the design models were used as an initial reference for building the final cryo-EM structures. For the hexamer the design model of the CalC6_3 was used as an initial reference model. For the heptamer, CalC6_3 with DHR extension was used as a starting model, as more of the DHR extension was visible in this map. A single chain for the heptamer was isolated in PyMOL and 7 copies of this chain were fitted to the map in UCSF Chimera^73^ to reform the channel. Hexamer and heptamer were refined using several rounds of relaxation and minimization, performed on the complete structures, which were manually inspected for errors each time using Isolde^74^, Coot^75,76^, and Phenix^77^ real-space refinement. The final model quality was analyzed using MolProbity^78^. Figures were generated using UCSF ChimeraX^79^. The final structures were deposited in the Protein Data Bank (PDB) under 9DZW, 9E0H, for the hexamer and heptamer respectively.

#### 2.13. Electrophysiology

We use the Bac-to-Bac system (Gibco) to express the designed channels in *Trichoplusia ni* insect cells (Hi5) for whole-cell patch-clamp experiments. The main advantage of this expression system is that cells expressing the designed proteins can be readily identified based on the morphological changes of Hi5 cells after being infected (characteristic features include an expansion of cell nuclei and an increase in cell granularity).

##### 2.13.1 Insect cell culture

SF9 cells and Hi5 cells (both from Thermo Fisher) were maintained at 27 °C and cultured in Grace’s Insect Medium supplemented with 10% fetal bovine serum (Avantor Seradigm 89510–186) and 1% penicillin-streptomycin-glutamine (Cytiva Hyclone). This medium was referred to as “supplemented medium” in the following sections.

##### 2.13.2. Baculovirus generation

The preparation of bacmid and baculovirus can also follow Invitrogen’s manual of Bac-to-Bac Baculovirus Expression System available online. We used a protocol that has been developed in the lab and previously used in published research. Designs in pFastBac-Dual vector were transformed into MAX Efficiency^™^ DH10Bac Competent Cells (Gibco). The transformed E. coli cells were then plated on LB agar plates supplemented with 50 μg/mL kanamycin, 7 μg/mL gentamicin, 10 μg/mL tetracycline, 100 μg/mL Bluo-Gal and 40 μg/mL IPTG and incubated at 37 °C for 48 hours. White colony was picked and transferred to 2 mL LB media supplemented with 50 μg/mL kanamycin, 7 μg/mL gentamicin and 10 μg/mL tetracycline to grow overnight. The cells were harvested by centrifugation at 14,000 g for 2 minutes and resuspended in 300 μL Solution 1 (15 mM Tris-HCl pH 8.0, 10 mM EDTA, 100 μg/ml RNaseA). 300 μL Solution 2 (0.2 M NaOH, 1% SDS) was added to this suspension, followed by gentle inversions several times and incubation at room temperature for 5 minutes to form clear lysates. 300 μL Solution 3 (potassium acetate, pH 5.5) was added to the lysate, followed by incubation on ice for 5 minutes. Cell debris was removed by centrifugation at 14,000 g for 10 minutes. 700 μL supernatant was mixed with 800 μL isopropanol and incubated on ice for 10 minutes. The mixture was centrifuged at 14,000 g for 10 minutes to pellet down the bacmid DNA. After the supernatant was discarded, the pellet was washed with 500 μL 70% ethanol, air dried at room temperature, and resuspended in 40 μL dH_2_O.

SF9 cells were seeded into 6-well tissue culture dishes at 50% confluency. Grace’s Insect Medium without serum or antibiotics (unsupplemented media) was used for transfection at this step. 10 μg bacmid DNA was added into 100 μL unsupplemented medium. 6 μL of Cellfectin II Reagent (Gibco) was diluted by 100 μL of unsupplemented medium and mixed with the bacmid DNA. The mixture was incubated at room temperature for 30 minutes. Pre-seeded SF9 cells were washed once with 2 mL unsupplemented medium. 800 μL of unsupplemented medium was added to the DNA-Cellfectin mixture and added to the cells. Media was exchanged to supplemented medium after incubation at 27 °C for 5 hours. The virus-containing supernatant was harvested 72 hours after transfection and was centrifuged at 500 g for 5 minutes to remove cell debris.

The virus (P1) was amplified once to generate a stable stock by addition to the SF9 cells cultured in the 150-mm tissue culture dish (at ~70% confluency). From the culture dish medium was aspirated, and virus suspension was gently added to the cells. The tissue culture dish was put on a rocker at room temperature for 1 hour, and incubated at 27 °C after addition of 25 mL medium. 48 hours after infection the virus-containing supernatant was harvested by centrifugation at 500 g for minutes and was stored at 4 °C (P2 virus).

##### 2.13.3. Western blot analysis

Western blot was used to confirm the expressions of designed channels. Hi5 cells were seeded into a 6-well tissue culture dish at 50% confluency. To infect the cells, 5 μL of the P2 virus was added to each well. The dish was put on a rocker at room temperature for 1 hour, and then incubated at 27 °C. Cells were harvested 48 hours after transfection, washed with 1 mL TBS buffer (20 mM Tris-HCl pH 8, 150 mM NaCl) supplemented with Pierce Protease Inhibitor Tablets, and resuspended in 60 μL TBS buffer. 5 μL of the cell suspension was added to 50 μL of the 2x Laemmli Sample Buffer (BIO-RAD) supplemented with 2-mercaptoethanol and loaded on gel. Proteins were transferred to the nitrocellulose membrane using the BIO-RAD Trans-Blot Turbo Transfer system. The membrane was incubated with 5% blotting grade blocker nonfat milk (BIO-RAD) in TBST (20 mM Tris-HCl, pH 8, 150 mM NaCl, 0.05% Tween 20) at 4 °C overnight. On the following day the membrane was washed three times with TBST and incubated with anti-His tag antibody conjugated with Horseradish Peroxidase (Jackson ImmunoResearch 300–035-240) in TBST (1:5000 dilution) for 1 hour. After antibody binding, the membrane was washed four times with TBST. Chemiluminescence was activated by BIO-RAD Clarity Western ECL Substrate and imaged using LI-COR Odyssey M Imager.

##### 2.13.4. Solution recipe for electrophysiology

All solutions used for electrophysiology were prepared from the stock solutions below:

NMDG-MeSO_3_ stock solution: 1.4 M NMDG, 200 mM HEPES, pH adjusted to 7.2 by methanesulfonic acid (~1.3 M).

NMDG-Cl stock solution: 1.4 M NMDG, 200 mM HEPES, pH adjusted to 7.2 by HCl (estimated Cl^−^ concentration of 1.3 M based on the added HCl volume).

NaMeSO_3_ stock solution: 1.4 M NaOH, 200 mM HEPES, pH adjusted to 7.2 by methanesulfonic acid (~1.3 M).

Stock solutions of divalent ions (Mg^2+^, Ca^2+^, and Sr^2+^) were prepared by mixing the corresponding hydroxides with MeSO_3_ to a final concentration of 100 mM for the metal ions. A little excess of MeSO_3_ was usually required to ensure complete dissolving of the metal hydroxides at a pH of 3~5. The pH of the solution was then brought back to 7.2 using the 1 M NMDG solution.

EGTA stock solution: 100 mM EGTA, 200 mM NMDG.

Extracellular solution: 140 mM NMDG, ~130 mM MeSO_3_, 20 mM HEPES, ~4 mM Cl^−^, x mM metal ions of interest, pH 7.2, osmolarity adjusted to 355 mmol/kg using sucrose.

Intracellular solution: 160 mM NMDG, ~110 mM MeSO_3_, 20 mM EGTA, 20 mM HEPES, ~10 mM Cl^−^, 4 mM MgATP, pH 7.2, osmolarity adjusted to 345 mmol/kg using sucrose.

pH was adjusted using the 1 M NMDG solution. Osmolarity was measured on a vapor pressure osmometer (VAPRO).

##### 2.13.5. Electrophysiology recording

Hi5 cells were seeded onto 10 mm glass coverslips (Ted-Pella, pre-cleaned with 70% ethanol) in 35 mm tissue culture dishes (GenClone 25–200). To express the designs in Hi5 cells, 2 μL of the P2 virus was added to the cells. The dish was put on a rocker at room temperature for 1 hour and then incubated at 27 °C. Whole-cell patch-clamp experiments were performed at 18–24 hours after infection. Patch pipettes were pulled from 4 mm thin-wall borosilicate glass capillaries (World Precision Instrument TW150F-4) using Sutter Model P-1000 micropipette puller and had a resistance of 4–7 MΩ when filled with the intracellular solution. Extracellular solutions were applied using a gravity-fed perfusion system equipped with a 8-channel perfusion manifold (Warner Instruments MPP-8), a flow regulating valve (Warner Instruments FR-50), and a cell-bathing chamber (Warner Instruments RC-25). The recording electrode was prepared from silver wires (A-M Systems, 0.008 inch, bare) through electrical chloriding in 3 M KCl solution. A pellet Ag/AgCl electrode (World Precision Instruments, EP2) was used as the reference electrode. Whole-cell currents were recorded using an EPC10 amplifier and PatchMaster Next software (Harvard Biosciences, version 1.2). Current signals were filtered at 2.9 kHz. Cells were patched in the 2 mM [Ca^2+^] extracellular solution. After breaking the patched cell membrane to establish the whole-cell configuration, the bath solution was changed to the 0.02 mM [Ca^2+^] solution. A −100 mV to +100 mV voltage-ramp protocol was applied every 5 seconds with a holding potential of 0 mV. In most cases the recorded currents using this ramp protocol were used to generate the I-V curves. The I-V curve obtained in the 0.02 mM [Ca^2+^] solution was used as the baseline for subtraction of leak current (Extended Data Fig. 7a). The currents measured at −100 mV were used for generating the time courses. After the currents elicited by the voltage-ramp protocol became stable, the bath solution was changed to the one containing the ions of interest (for example, a solution containing 10 mM [Ca^2+^] was used to determine the Ca^2+^ conductance). For measurement of channel conductance for different ions, the 0.02 mM [Ca^2+^] solution was used between the solution exchange to remove the previous ion. Liquid junction potentials between the extracellular and intracellular solutions were calculated to be less than 1 mV (https://swharden.com/LJPcalc/) and therefore was not corrected for the command voltage. The cell capacitance was measured using the automated capacitance correction function of EPC10/Patchmaster Next. Data was exported as ASCII files and analyzed using Python.

##### 2.13.6. Methods for comparing relative conductances of designed channels for different ions

We use Goldman–Hodgkin–Katz (GHK) flux equation to compare the relative conductances of designed channels for different ions based on the measured inward current at −100 mV:

(1) $$\Phi_{s}=P_{s}\cdot z_{s}^{2}\cdot \frac{V_{m}F^{2}}{RT}\cdot \frac{{\left[S\right]}_{i}-{\left[S\right]}_{o}exp\left(-z_{s}V_{m}F/RT\right)}{1-exp\left(-z_{s}V_{m}F/RT\right)}$$

where $\Phi_{s}$ is the ion flux (i.e., the measured current or current density), $P_{s}$ is the permeability for ion S, $z_{s}$ is the valence of ion S, $V_{m}$ is the membrane potential, F is the Faraday constant, R is the gas constant, T is the absolute temperature, and ${\left[S\right]}_{i}$ and ${\left[S\right]}_{o}$ are the intracellular and extracellular concentrations of ion S, respectively.

Given that we are comparing the inward currents and assume that there is no ion of interest inside the cell (${\left[S\right]}_{i}=0$), equation (1) is then simplified to:

(2) $$\Phi_{s}=-P_{s}\cdot z_{s}^{2}\cdot \frac{V_{m}F^{2}}{RT}\cdot \frac{{\left[S\right]}_{o}exp\left(-z_{s}V_{m}F/RT\right)}{1-exp\left(-z_{s}V_{m}F/RT\right)}$$

To compare the permeability of two different ions (*S*_*1*_ and *S*_*2*_), Equation (2) can be rewritten as:

(3) $$\frac{P_{s,1}}{P_{s,2}}=\frac{\Phi_{s,1}}{\Phi_{s,2}}\cdot \frac{z_{s,2}^{2}}{z_{s,1}^{2}}\cdot \frac{1-exp\left(-z_{s,1}V_{m}F/RT\right)}{1-exp\left(-z_{s,2}V_{m}F/RT\right)}\cdot \frac{{\left[S_{2}\right]}_{o}exp\left(-z_{s,2}V_{m}F/RT\right)}{{\left[S_{1}\right]}_{o}exp\left(-z_{s,1}V_{m}F/RT\right)}$$

Since in most cases the currents at −100 mV are compared (*V*_*m*_ ≪ 0), equation (3) can be simplified to:

(4) $$\frac{P_{s,1}}{P_{s,2}}=\frac{\Phi_{s,1}}{\Phi_{s,2}}\cdot \frac{z_{s,2}^{2}}{z_{s,1}^{2}}\cdot \frac{{\left[S_{2}\right]}_{o}}{{\left[S_{1}\right]}_{o}}$$

To directly compare the relative conductance of the channels for each individual ion using the measured current or current density, the following condition needs to be satisfied:

(5) $$\frac{z_{s,2}^{2}}{z_{s,1}^{2}}\cdot \frac{{\left[S_{2}\right]}_{o}}{{\left[S_{1}\right]}_{o}}=1$$

Equation (5) can be written as:

(6) $$\frac{{\left[S_{1}\right]}_{o}}{{\left[S_{2}\right]}_{o}}=\frac{z_{s,2}^{2}}{z_{s,1}^{2}}$$

Therefore, the ion concentration used for determining the relative conductances for different ions was inversely proportional to the square of valence of the particular ion. As a result, the concentration of divalent ions was determined to be 10 mM, and the concentration of Na^+^ was determined to be 40 mM.

### 2.14. NFAT-RE-luciferase expression assay

HEK293T cells were seeded into a white 96-well plate (Greiner Bio-One #655088) pre-treated with poly-L-lysine (Sigma-Aldrich P4707) and cultured in DMEM (with 10% FC-II and 1% penicillin-streptomycin). CalC6_3 and its E101L mutant were cloned into the pcDNA3.1(+) vector in the format of channel-P2A-mScarlet3. The plasmid encoding the channels was co-transfected with the pGL4.30 [luc2P/NFAT-RE/Hygro] plasmid (Promega, Madison, WI, USA) using the jetPRIME transfection reagent (Sartorius Polyplus #101000015) following manufacturer’s instructions. The psPAX2 plasmid (Addgene #12260), which expresses proteins unrelated to Ca^2+^ flux or buffering, was used as the negative control in this experiment. The culture medium was exchanged into DMEM (with 10% FC-II and 1% penicillin-streptomycin) 24 hours after transfection. At 48 hours post-transfection, cells were washed twice with FluoroBrite DMEM (Gibco #A1896701, with 10% FC-II) and incubated in 50 μL of this FluoroBrite DMEM medium. mScarlet3 fluorescence was measured immediately before addition of the luciferase substrate for normalization of the expression level of the designs. The Bright-Glo Luciferase Assay System (Promega E2610) was used to measure the luminescence from the cells. 50 μL of the reconstituted Bright-Glo Luciferase Assay Substrate (in Bright-Glo Luciferase Assay Buffer) was added to each well of the microplate, and luminescence was measured for 30 minutes using the BioTek Synergy Neo2 plate reader. The maximum luminescence intensity during the measurement was used for plotting.

### 3. Visualization and figures

All structural images and maps were generated using PyMOL (v3.0.4) or ChimeraX (v1.7.0)^79^. Cartoon representations, design schematics, and experimental diagrams were prepared using Inkscape.

## Extended Data

**Extended Data Fig. 1:**
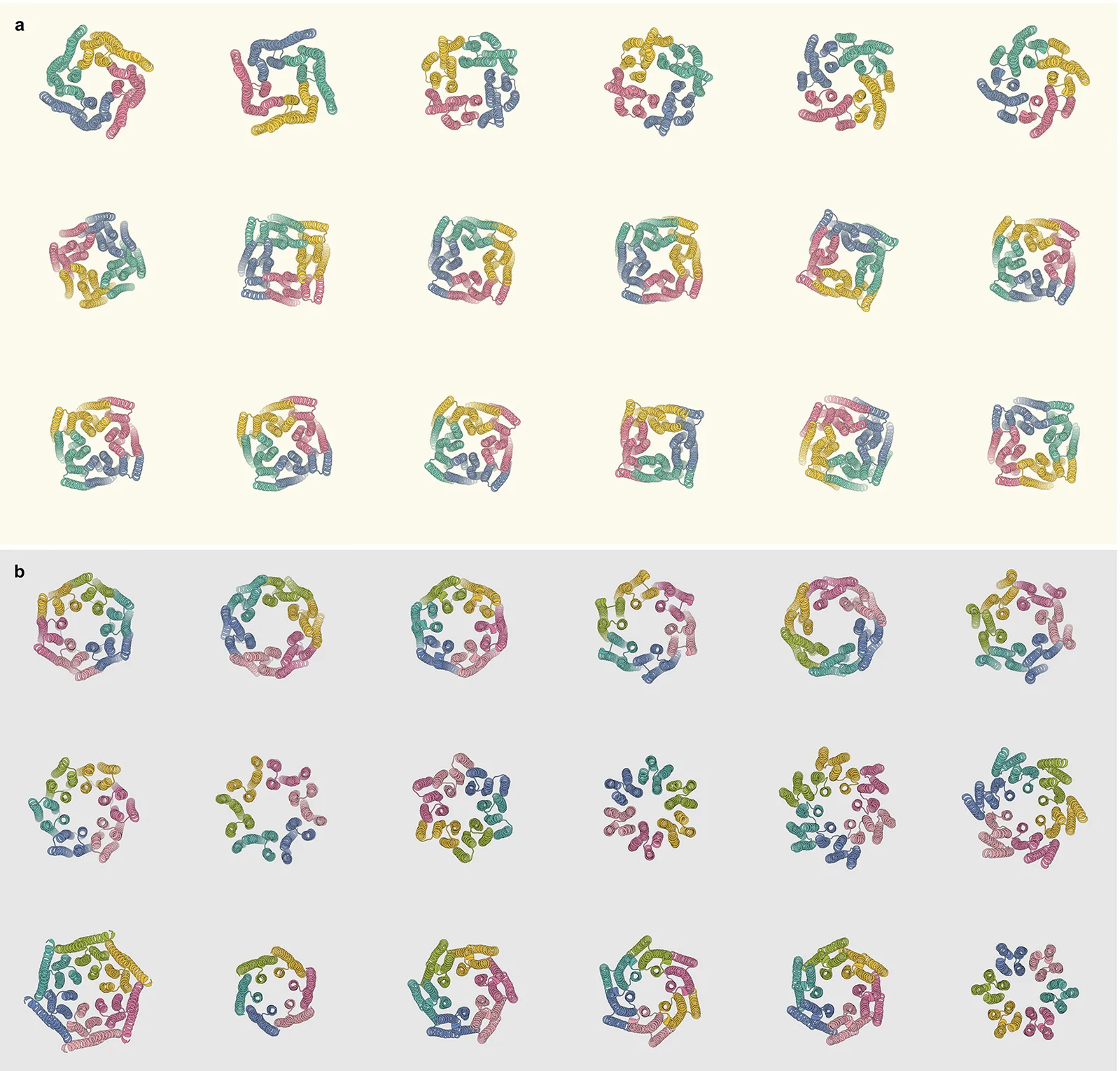
Examples of AlphaFold2-predicted structures of designed Ca^2+^channels with pTM scores higher than 0.8. **a,** Designs with C4 symmetry. **b,** Designs with C6 symmetry.

**Extended Data Fig. 2:**
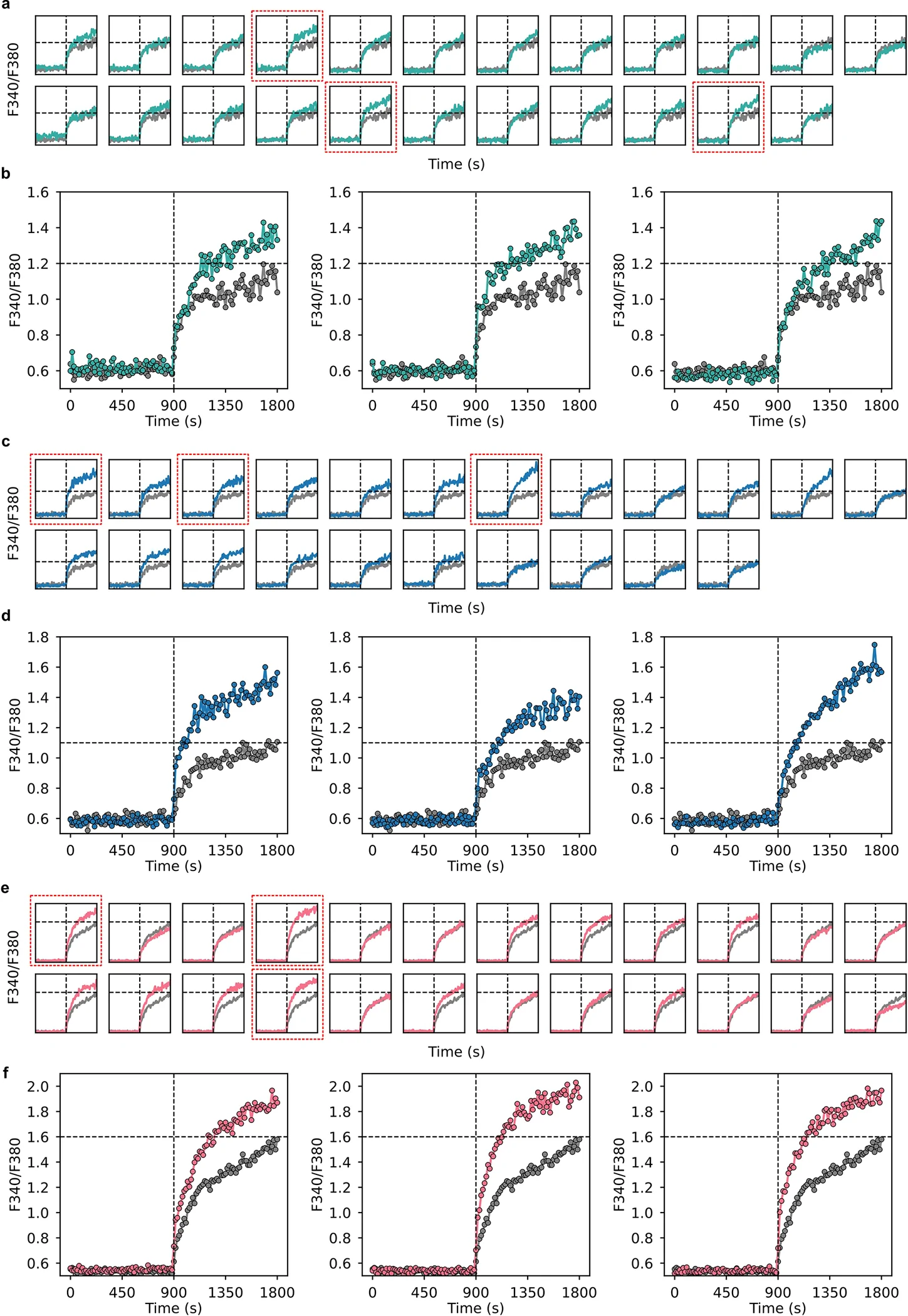
Flux assay in HEK293T cells using Fura-2 AM. **a-b** (cyan), **c-d** (blue), and **e-f** (red) were data for CalC4, CalC6, CalC6_H designs, respectively. Ba^2+^ ions were added at the 900-second time point indicated by the vertical dashed lines. The gray traces represented measurements from mock-transduced cells as the negative control and were overlaid with the colored traces representing the cells transduced with the designs in each plot for comparison. **b**, **d** and **f** were close-up views of the plots highlighted by the red dashed frames in **a**, **c** and **e,** respectively, which were examples that were identified as the putative designs manifesting divalent ion permeability.

**Extended Data Fig. 3:**
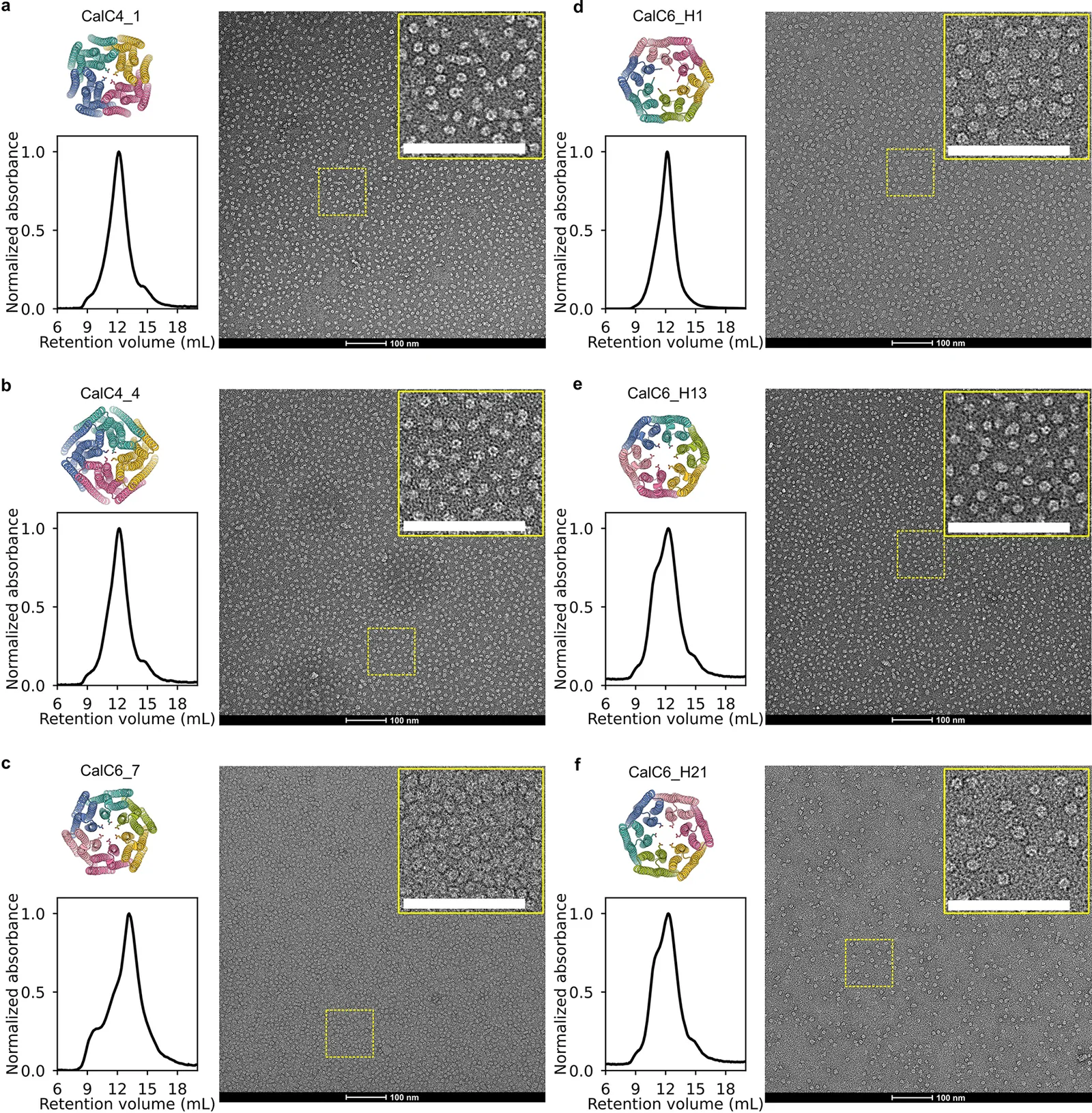
Biophysical characterizations of more designed channels. In addition to the three designs shown in Fig. 2, two more CalC4 designs, CalC4_1 **(a)** and CalC4_4 **(b)**, one more CalC6 design, CalC6_7 **(c)**, and three more CalC6_H designs, CalC6_H1 **(d)**, CalC6_H13 **(e)**, and CalC6_H21 **(f)**, were observed to elute as desired oligomeric states from SEC and assemble into homogeneous pore-containing particles on the ns-EM grids. The close-up views shown on the upper right corners of the ns-EM micrographs corresponded to the regions highlighted by the dashed yellow frames. Scale bars, 100 nm.

**Extended Data Fig. 4:**
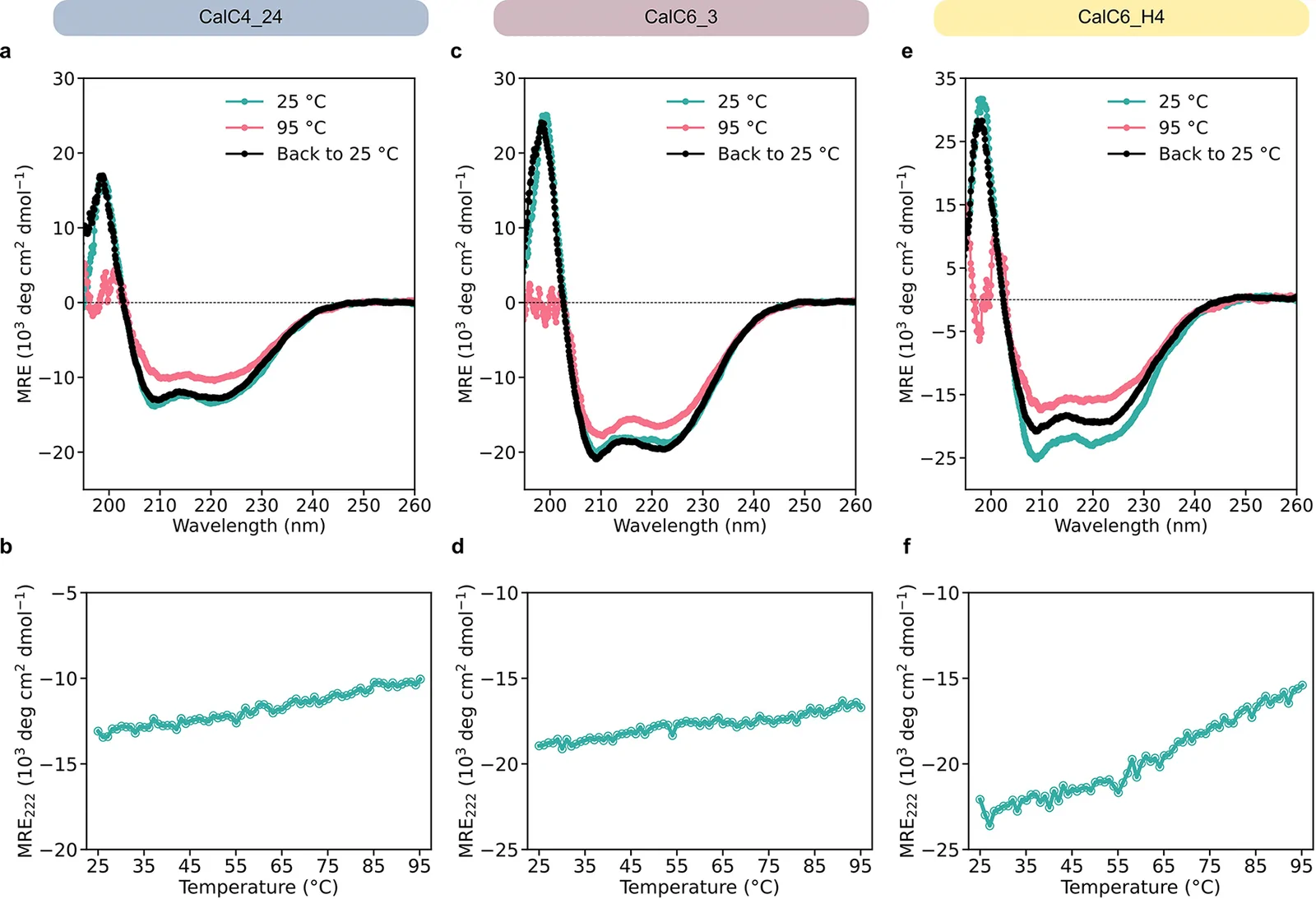
Circular dichroism results of CalC4_24 (**a-b**), CalC6_3 (**c-d**), and CalC6_H4 (**e-f**). **a**, **c**, and **e**, CD spectra at 25 °C (cyan lines), 95 °C (red lines), and cooled back to 25 °C (black lines). **b**, **d**, and **f**, CD melting curves at 222 nm.

**Extended Data Fig. 5:**
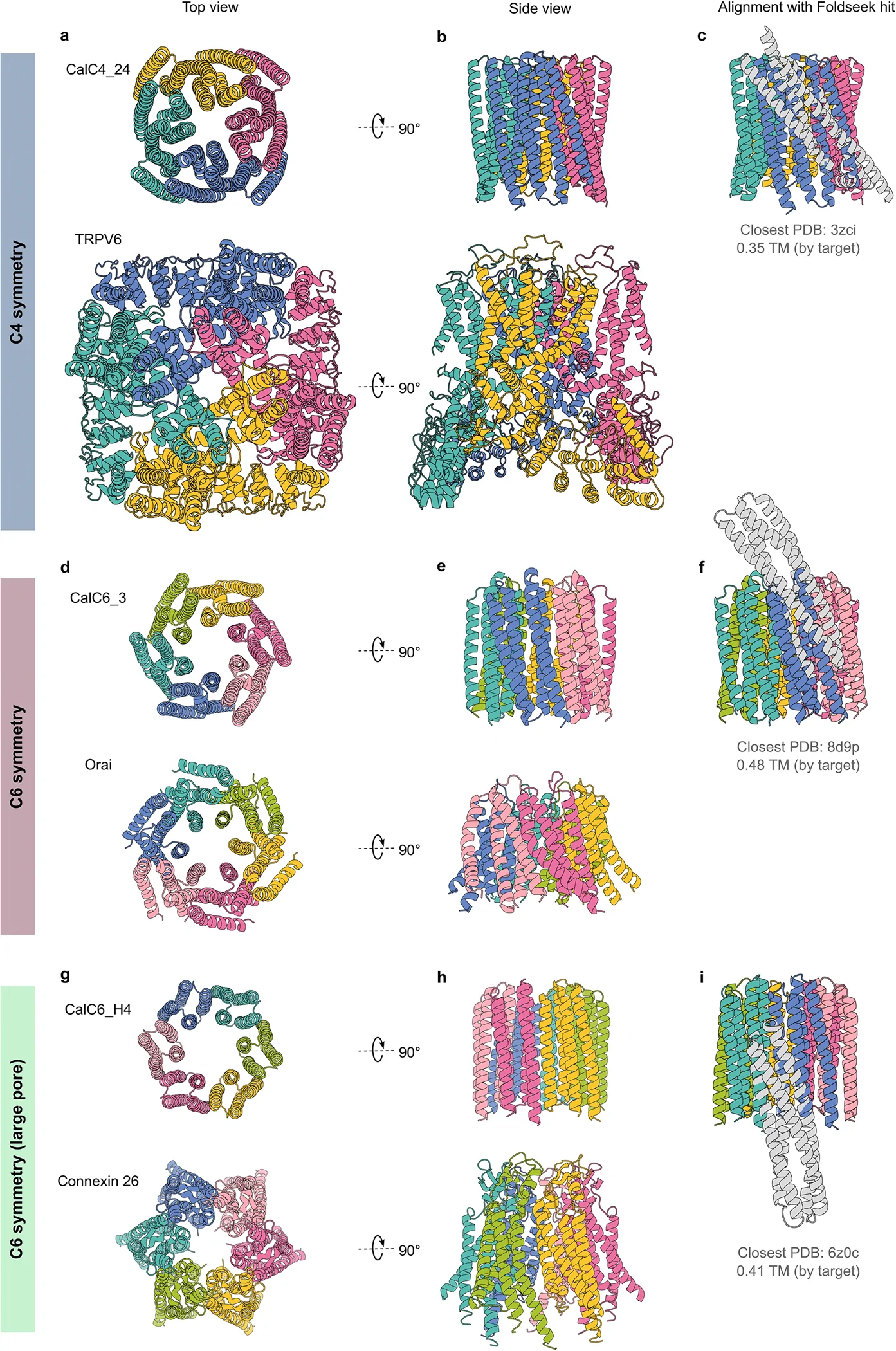
Comparison between three representative designed channels and native channels, as well as the closest structures in the PDB (by Foldseek). CalC4_24 was compared with TRPV6 (PDB 6BO8), CalC6_3 was compared with Orai (PDB 7KR5), and CalC6_H4 was compared with connexin 26 (PDB 2ZW3) from the top (**a**, **d**, and **g**) and the side view (**b**, **e**, and **h**), respectively. **c**, **f**, and **i**, Overlay of CalC4_24 (**c**), CalC6_3 (**f**), and CalC6_H (**i**) and their closest structures in the PDB (3zci in **c**, TM score=0.35; 8d9p in **f**, TM score=0.48; 6z0c in **i**, TM score=0.41).

**Extended Data Fig. 6:**
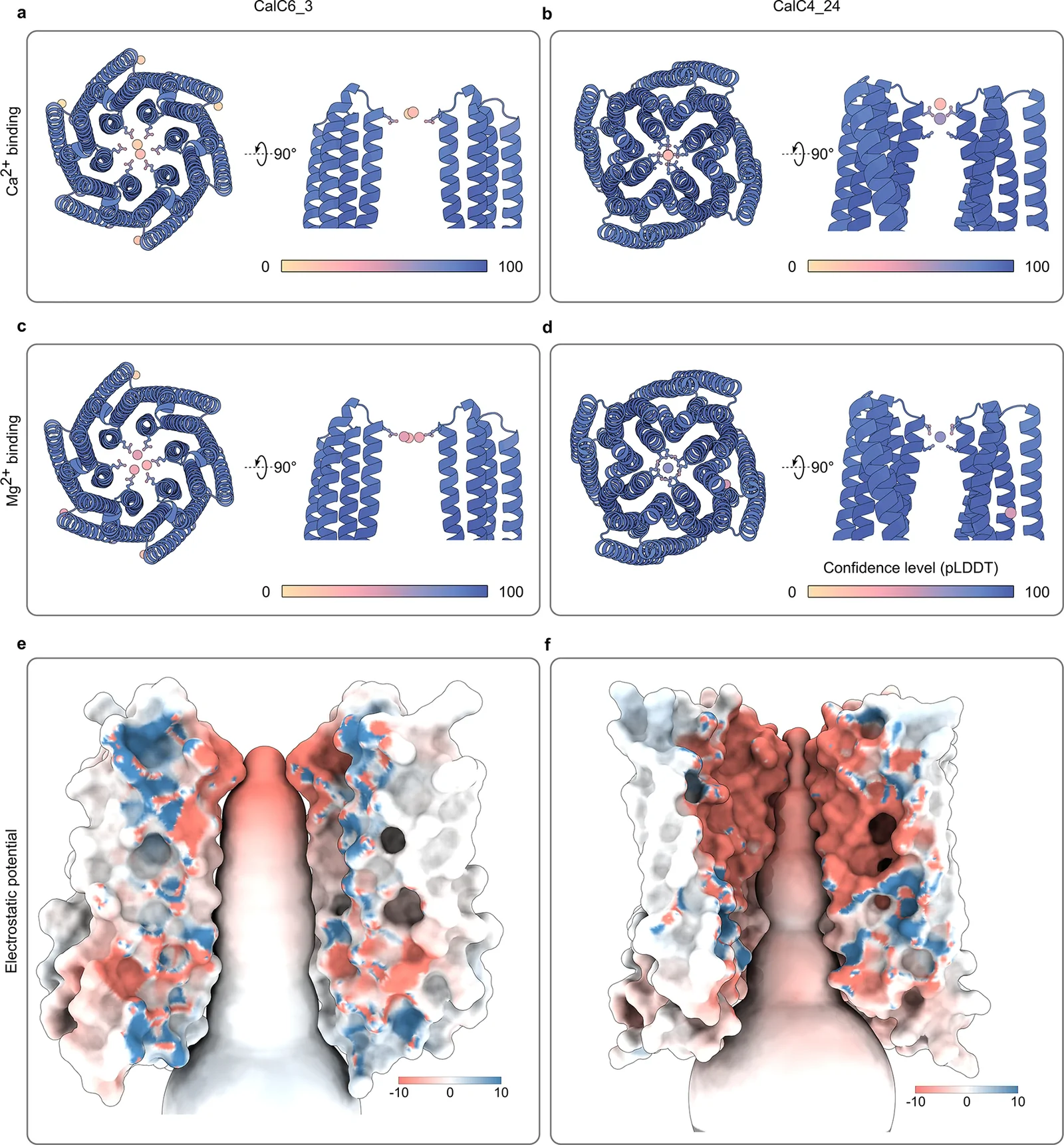
Predicted biophysical properties of designed channels. **a-d**, AlphaFold3-predicted binding of CalC6_3 and CalC4_24 to Ca^2+^ (**a** and **b**) and to Mg^2+^ (**c** and **d**), respectively. The models were colored based on the confidence level (pLDDT) of the prediction results, with the color gradient shown on the bottom right. **e** and **f**, calculated electrostatic potential of the protein surfaces and the ion permeation pathways of CalC6_3 (**e**) and CalC4_24 (**f**). For clarity only the opposing chains of the designed channels are shown. The ion permeation pathways are calculated using MoleOnline. The electrostatic potential is calculated using the Adaptive Poisson-Boltzmann Solver (APBS) in PyMOL.

**Extended Data Fig. 7:**
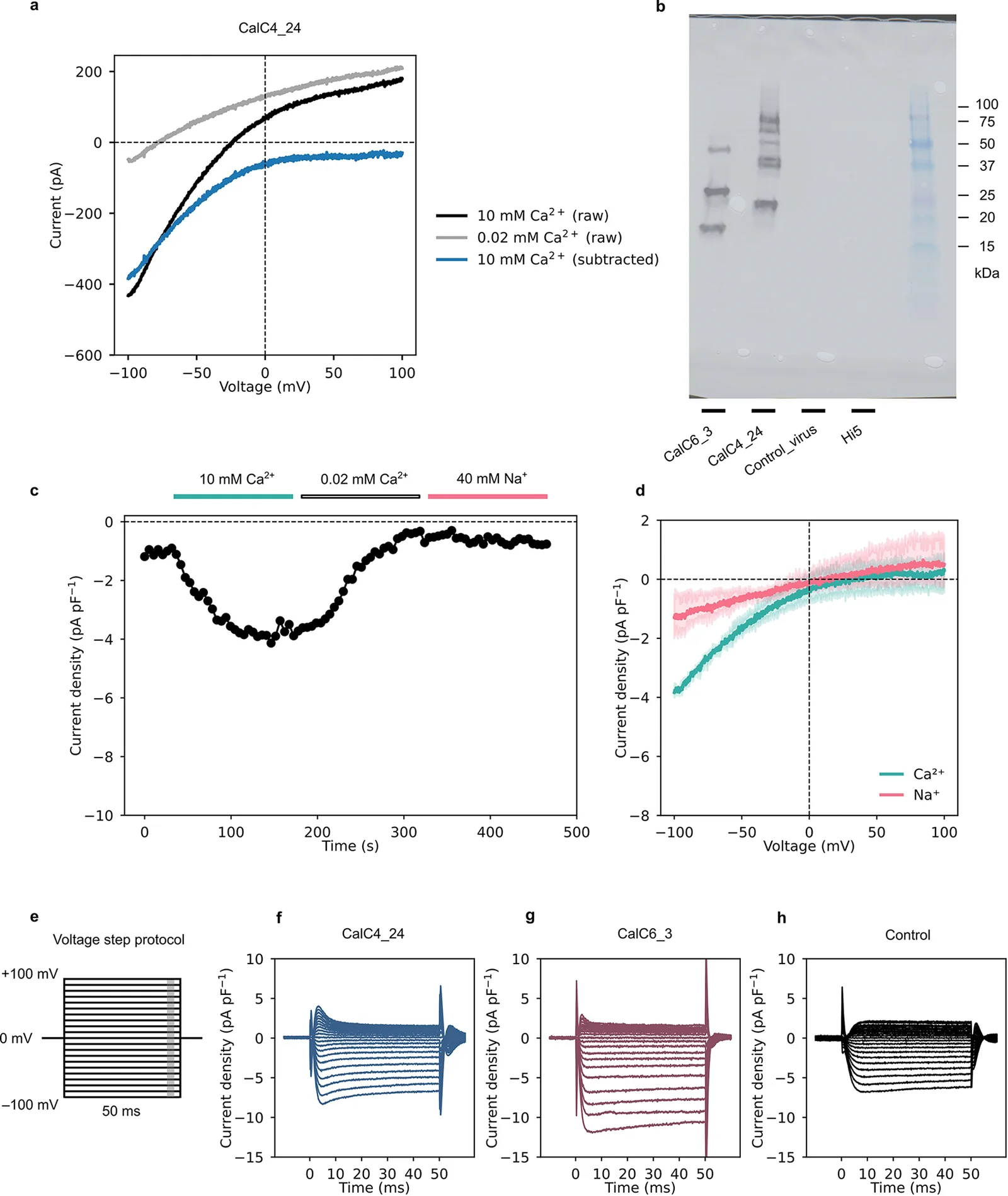
Additional characterizations of designs and negative controls. **a**, An example of I-V curve plot demonstrating the data processing method. The black curve represented the raw data obtained from a −100 mV to +100 mV ramp protocol in 10 mM [Ca^2+^] solution. The gray curve represented the raw data obtained using the same ramp protocol in 0.02 mM [Ca^2+^] solution, and was defined as the background. The blue curve was obtained by subtracting the gray curve from the black curve, and was defined as the Ca^2+^ conductance. The same process was used to determine conductances for other ions as well. **b**, Expression of designs in Hi5 cells examined by western blot using an anti-His antibody. The four samples loaded on the gel (lanes indicated by the black bars) were whole-cell lysates of cells expressing CalC6_3 and CalC4_24, cells infected with the control virus (made from an empty pFastBac_Dual vector), and uninfected cells alone. **c,** The time course of currents at −100 mV in 10 mM [Ca^2+^] and 40 mM [Na^+^] extracellular solutions recorded on cells infected with the control virus. **d**, I-V relations obtained from a −100 mV to +100 mV ramp protocol in 10 mM [Ca^2+^] (cyan) and 40 mM [Na^+^] (red) solutions, averaged over three separate cells infected with the control virus. The shaded regions represent bootstrapped 95% confidence intervals. **e-h,** Comparison of Ca^2+^ current densities elicited by the voltage step protocol in the 10 mM [Ca^2+^] extracellular solution between the designs and the negative control (without subtraction of background leak currents). **e,** The schematic illustration of the voltage step protocol (also shown in Fig. 3). **f** and **g**, Current densities obtained from the cells expressing CalC4_24 (**f**) and CalC6_3 (**g**). The source data are the same as those shown in Fig. 3e and f. Here, the currents are normalized by cell capacitances to obtain current densities. **h**, Current densities obtained from the cell treated with the control virus.

**Extended Data Fig. 8:**
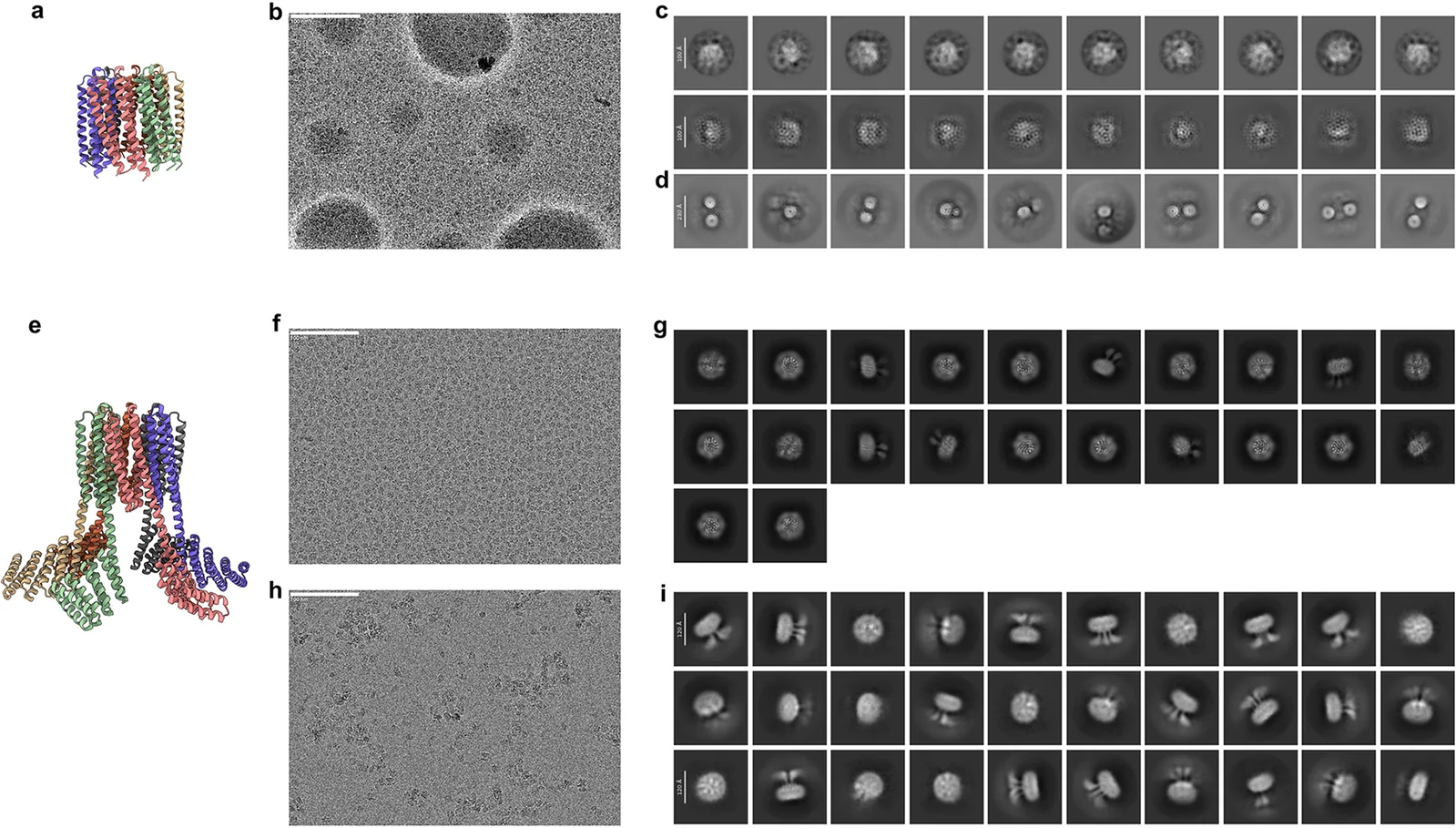
Cryo-EM micrographs and 2D averages for CalC6_3 before and after DHR extensions. **a-d**, Design model **(a)**, a representative cryo-EM micrograph of protein particles on a lacey carbon grid **(b)**, and 2D class averages (**c-d**) from three different 2D classification settings, with an extraction box size of 300 pixels used in **c** and an extraction box size of 680 pixels (pixel size 0.4135 Å) used in **d**. **e-i**, Design model **(e)** and cryo-EM data **(f-i)** for CalC6 with DHR extensions. **f** and **h**, Representative cryo-EM micrographs of protein particles on a thin carbon grid **(f)** and a holey carbon grid **(h)**, respectively. **g** and **i**, 2D class averages from the thin carbon grid **(g)** and from the holey carbon grid **(h)**, respectively. Scale bars in the micrographs, 100 nm. Scale bars in **c**, 10 nm. Scale bar in **d**, 23 nm. Scale bars in **i**, 12 nm.

**Extended Data Fig. 9:**
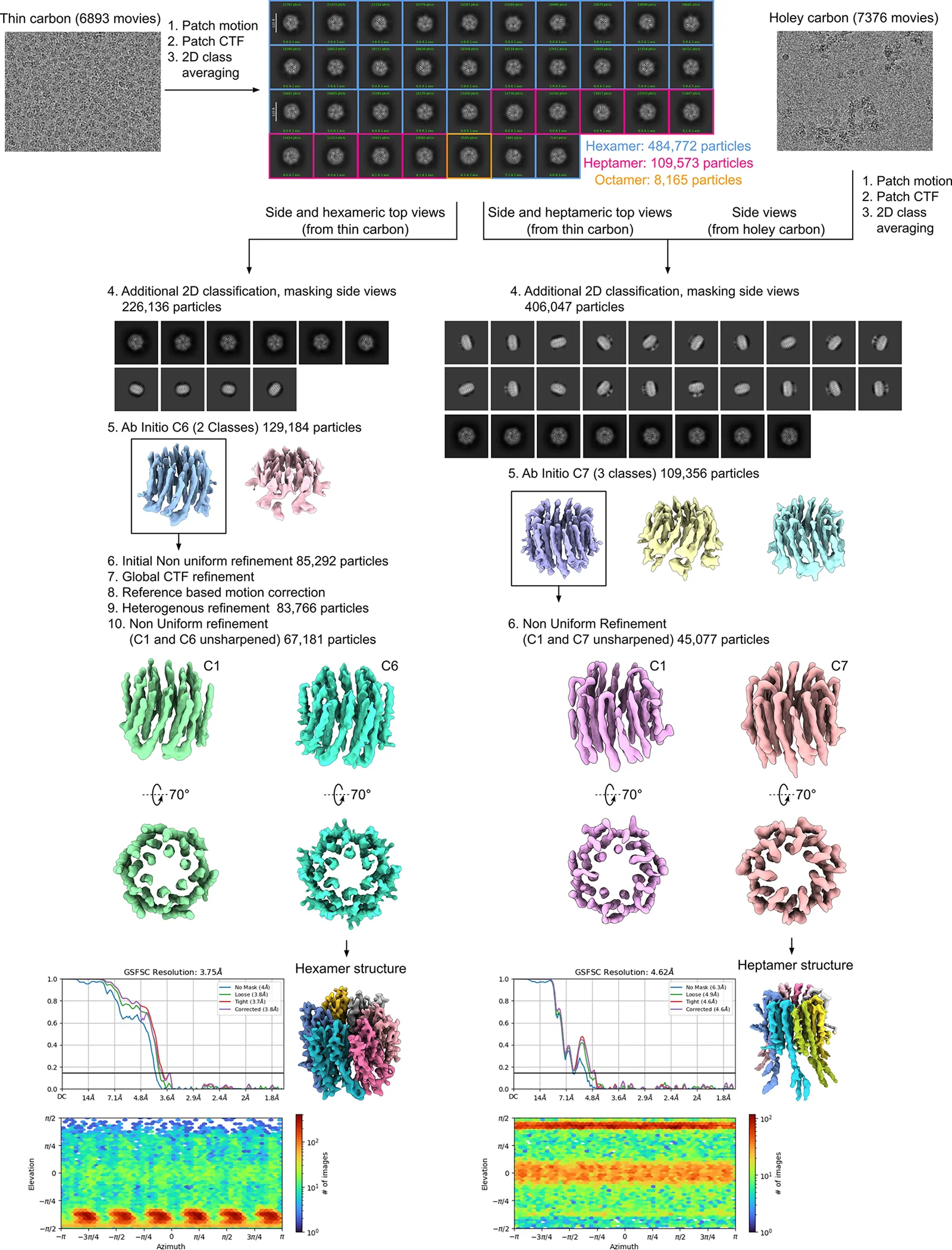
Workflow for cryo-EM data processing of the CalC6_3 design with DHR extensions. The flowchart demonstrated particle picking, classification, reconstruction and refinement processes that enabled determination of both a hexamer and a heptamer structure. All data processing steps were carried out in CryoSPARC v4.4. Detailed processes can be found in Methods (2.11 and 2.12).

**Extended Data Fig. 10:**
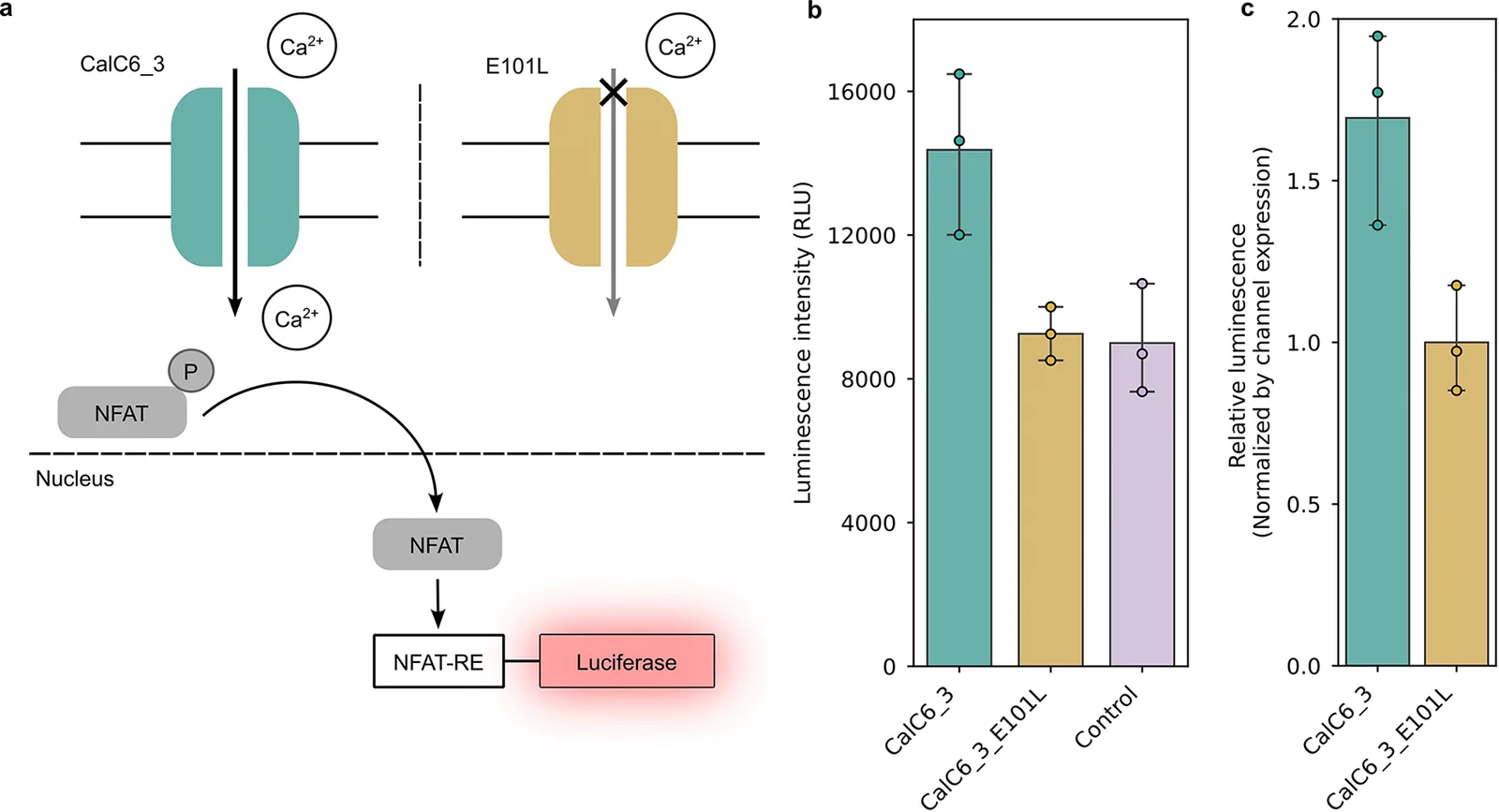
NFAT-RE-luciferase expression assay. **a**, Schematic diagram of the assay. Ca^2+^ influx through the designed channel in HEK293T cells would induce expression of the luciferase reporter through the Ca^2+^-dependent NFAT signaling pathway. **b** and **c**, Bar plots showing the average maximum luminescence intensity measured from cells (n=3) expressing the wild-type CalC6_3 design (cyan), the E101L mutant (yellow), and the negative control (purple), respectively. The channel constructs contain a C-terminal mScarlet3 linked to the channel via the P2A self-cleaving sequence. The psPAX2 plasmid, which expresses proteins unrelated to Ca^2+^ flux or buffering, was used as the negative control. **b**, Raw luminescence intensity. **c**, Luminescence intensity normalized by mScarlet3 fluorescence. Error bars represent bootstrapped 95% confidence intervals around the mean.

**Extended Data Table 1:** Cryo-EM data collection and processing parameters for CalC6_3 with DHR extension domains.

Data Collection		
Microscope	Titan Krios	
Voltage (kV)	300	
Detector	Gatan K3	
Energy Filter	Gatan BioQuantum Gif	
Recording mode	Counting	
Magnification	105,000 X	
Movie micrograph pixel size (Å)	0.843	
Dose rate (e^−^/Å^2^/s)	11.83	
No. of frames per movie micrograph	79	
Frame exposure time (s)	0.0506	
Movie micrograph exposure time (s)	4.0	
Total dose (e^−^/Å^2^)	47.32	
Under focus range (μm)	0.8–1.8	
Number of movie micrographs	14,269	
**Map Processing**	Hexamer	Heptamer
Extraction Box Size (pix)	340	340
Initial particle images (no.)	886,110	886,110
Final particle images (no.)	67,181	45,077
Map resolution (Å)	3.75	4.62
FCS threshold	0.143	0.143
Map resolution range (Å)	3.38–4.56	4.20–8.80
**Refinement**		
Initial model used	Design Model	Design Model
Map resolution (Å)	3.75	4.62
FCS threshold	0.143	0.143
Model resolution range	3.38–4.56	4.20–6.5
Map sharpening B factor	Na	343.8
Model composition		
Non-hydrogen atoms	6,215	6,069
Protein Residues	846	1,057
Ligands	0	0
*B* factors (Å)		
Protein	Na	249.89
Ligands	Na	–
R.M.S. deviations		
Bond lengths (Å)	0.012	0.001
Bond angles (^o^)	1.795	0.369
Validation		
MolProbity score	1.00	0.69
Clashscore	0.78	0.57
Rotamer Outliers (%)	2.13	0.00
Ramachandran plot		
Favored (%)	98.32	99.33
Allowed (%)	1.68	0.67
Disallowed (%)	0.00	0.00

## Supplementary Material

Supplementary information

## Figures and Tables

**Fig. 1:**
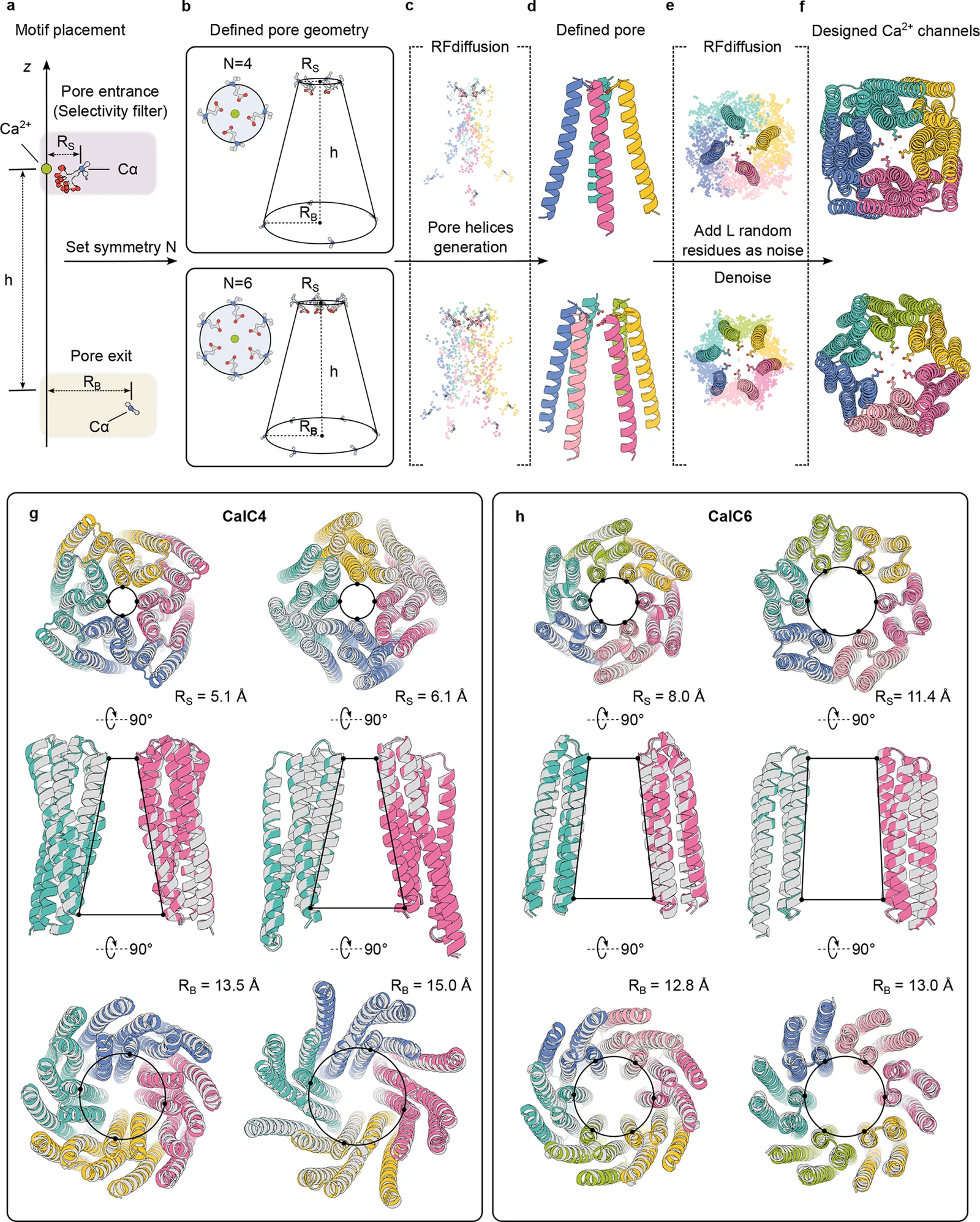
A general approach for designing Ca^2+^ channels from selectivity filter geometry. **a,** A pair of Ca^2+^ ion (green sphere) and an acidic amino acid (Glu in this case, with the C-alpha atom colored in blue) and an additional pore exit defining residue (only backbone atoms shown) are used as the initial structural motifs. The distance between the Ca^2+^ ion and the C-alpha atom (R_s_) defines the size of the selectivity filter regardless of the specific rotamer conformations of the Glu residue. The pore exit defining residue is positioned at a distance (h) along the z-axis from the Glu residue; the distance from the C-alpha atom of this residue to the z-axis (R_B_) defines the radius of the pore exit. **b,** Application of N-fold cyclic symmetry generates the framework of the pore defined by R_s_, R_B_, h and symmetry (N), and the selectivity filter coordinating the Ca^2+^ ion. **c and d,** The selectivity filter and pore exit defining residues are connected by helices using RFdiffusion. **e and f,** The pore-lining helices are extended to form channel proteins using RFdiffusion by adding noise consisting of L residues in each monomer followed by a denoising trajectory. **g and h,** Examples of designed channels (colored models) aligned with AlphaFold2 predicted models (gray) from the top view (top), side view (middle) and bottom view (bottom). For side views only the opposing two chains are shown for clarity. **g**, Two designs with C4 symmetry (CalC4), with backbone RMSD values of 0.8 Å and 0.2 Å to AlphaFold2 predictions, respectively. **h,** Two designs with C6 symmetry (CalC6), with backbone RMSD values of 0.8 Å and 0.5 Å to AlphaFold2 predictions, respectively.

**Fig. 2:**
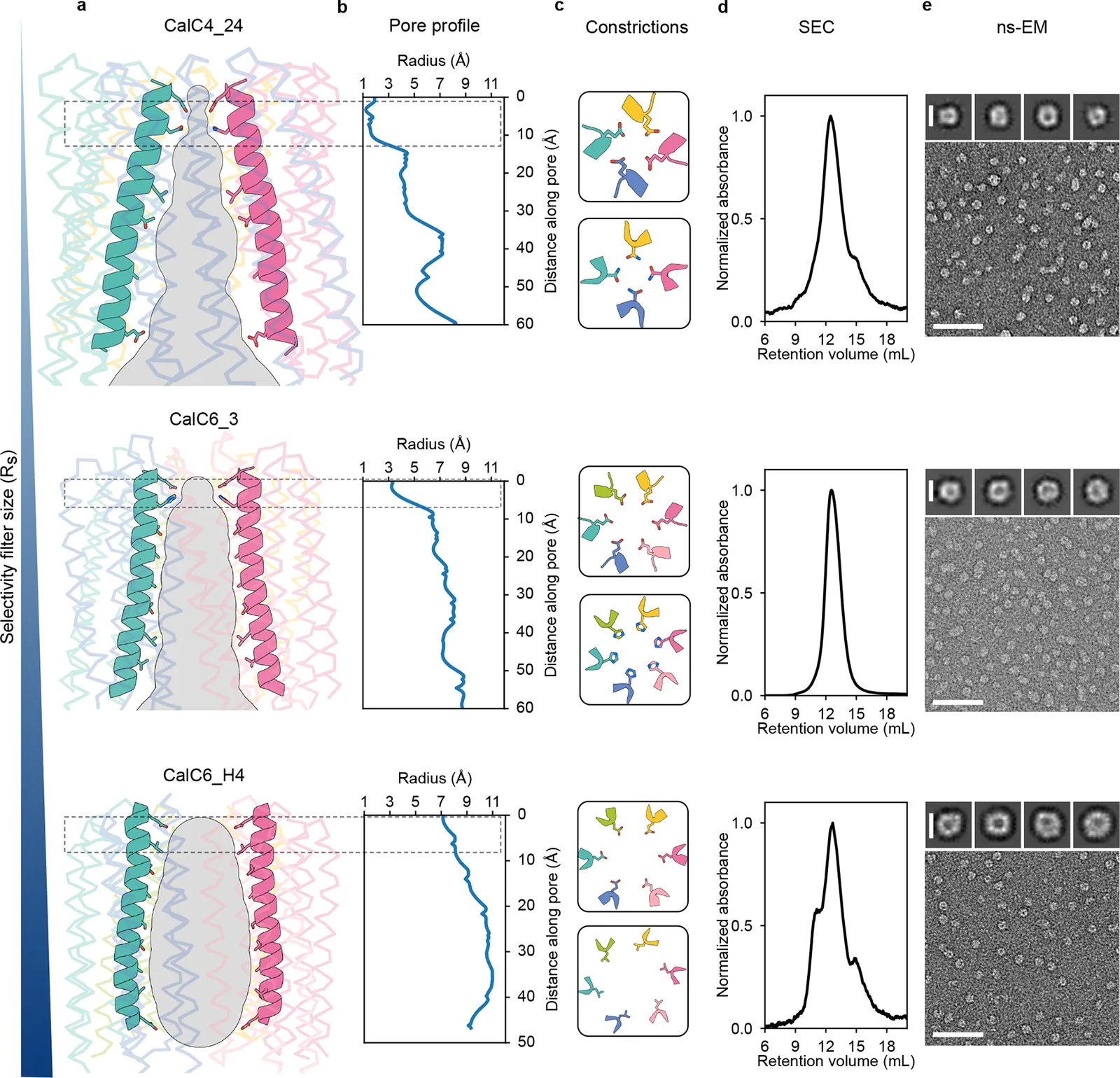
Biophysical characterization of designed Ca^2+^ channels. **a,** Side views of the designed Ca^2+^ channels. Ion permeation pathway and the pore-lining helices on two opposing chains are highlighted, with the remainder of the channels rendered in transparency for clarity. **b,** Pore radius profiles representing the ion permeation pathways in **(a)**. **c,** Top views of the constrictions formed by the sidechains of the residues on two consecutive helical turns. The constrictions are located at the pore entrance, shown by the dashed frame in **(a)** and **(b)**. **d,** Size-exclusion chromatography traces of the purified Ca^2+^ channels. **e,** Representative ns-EM micrographs (scale bars, 50 nm) and 2D class averages (scale bars, 8 nm) of the designed channels.

**Fig. 3:**
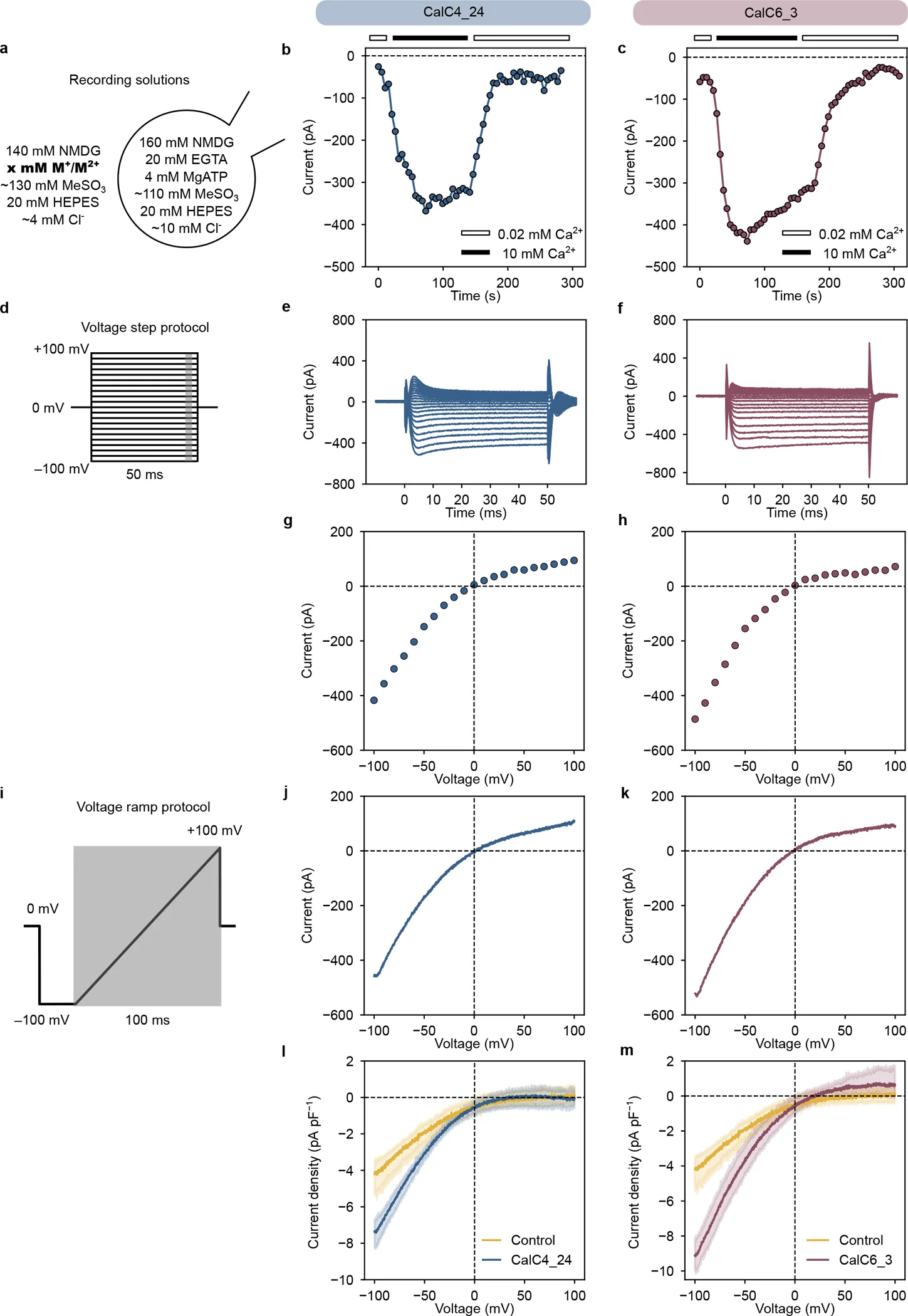
Ca^2+^ conduction by the designed channels. **a,** Diagram showing the solutions used for whole-cell patch-clamp recordings. M^+^ denotes monovalent ions and M^2+^ denotes divalent ions. **b** and **c,** Time courses of the inward whole-cell currents of CalC4_24 **(b)** and CalC6_3 **(c)** recorded at −100 mV in Hi5 cells. Cells were first bathed in 20 μM [Ca^2+^] NMDG-based solution (the first unfilled bars). The extracellular solution was then changed to 10 mM [Ca^2+^] solution (the black bars), and was finally changed back to 20 μM [Ca^2+^] solution (the third unfilled bars). **d,** The schematic illustration of the voltage step protocol. **e** and **f**, Ca^2+^ currents elicited by the voltage step protocol recorded on Hi5 cells expressing CalC4_24 (**e**) and CalC6_3 (**f**), respectively. **g** and **h**, the I-V relations obtained from **e** and **f**, respectively. Tail currents (shown as the gray region in **d**) were used to plot the I-V curves. **i,** The schematic illustration of the voltage ramp protocol. The gray region shows the range used for plotting the I-V curves. **j** and **k**, Ca^2+^ currents elicited by the voltage ramp protocol recorded on Hi5 cells expressing CalC4_24 (**j**) and CalC6_3 (**k**), respectively. **l** and **m**, Current densities from cells expressing CalC4_24 (**l**) and CalC6_3 (**m**) in 10 mM [Ca^2+^] solution after background subtraction using currents recorded in 0.02 mM [Ca^2+^] solution. The control curves (yellow) were obtained from cells infected with the baculovirus that did not encode recombinant proteins (made from an empty pFastBac-Dual plasmid), and were included in both **l** and **m** for comparison. Each I-V curve in **l** and **m** was obtained by averaging over four measurements on separate cells. The shaded regions represent bootstrapped 95% confidence intervals.

**Fig 4:**
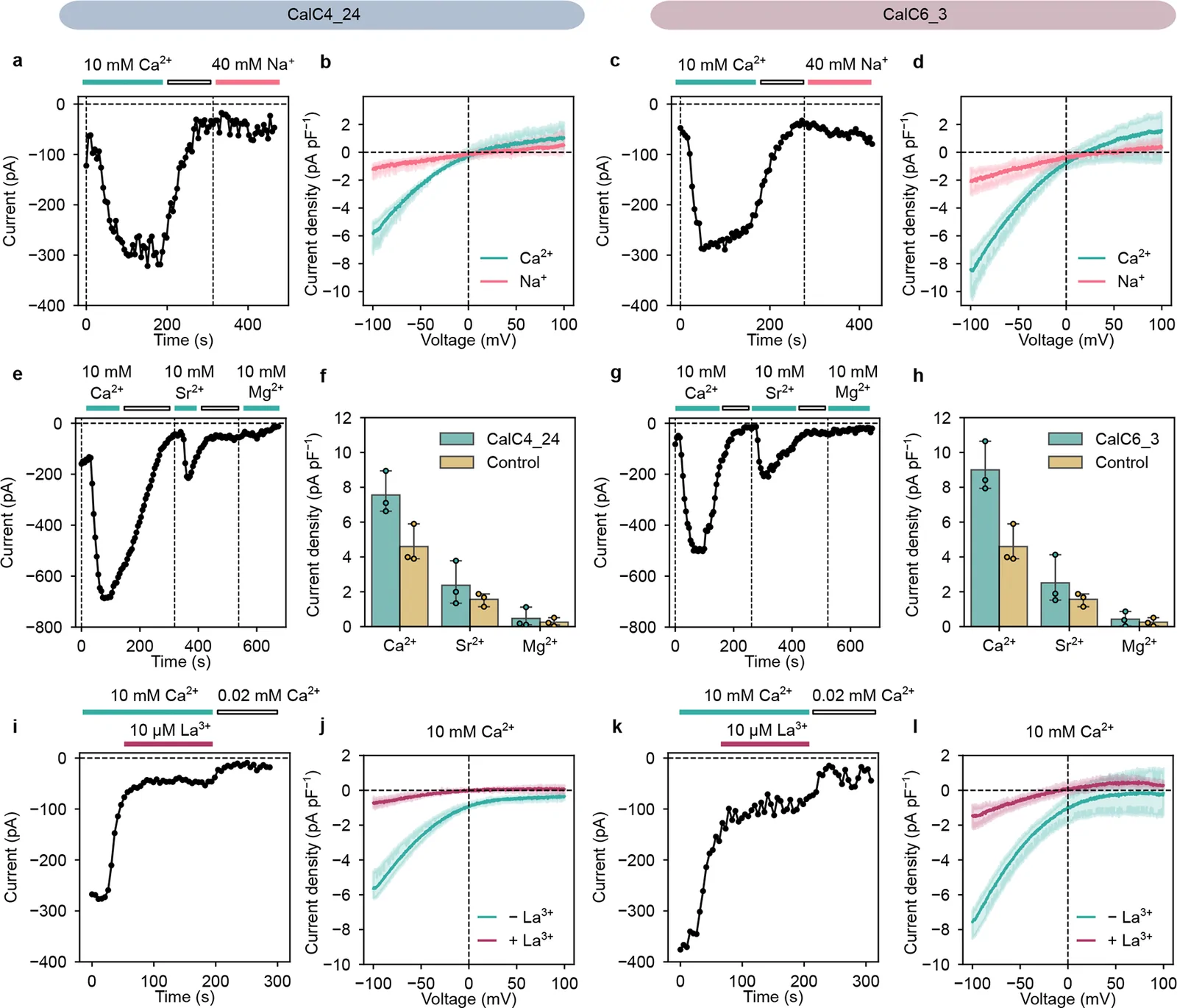
Ion selectivity of designed Ca^2+^ channels. **a-d,** Relative conductances of CalC4_24 **(a-b)** and CalC6_3 **(c-d)** for Ca^2+^ and Na^+^. **a** and **c**, Time courses of currents at −100 mV. Extracellular solution was exchanged from a solution containing 10 mM [Ca^2+^] to 40 mM [Na^+^] with 0.02 mM [Ca^2+^] solution between the exchange. **b** and **d**, I-V relations obtained from a −100 mV to +100 mV ramp protocol (Ca^2+^ currents in cyan and Na^+^ currents in red). **e-h,** Relative conductances of CalC4_24 **(e-f)** and CalC6_3 **(g-h)** for Ca^2+^, Sr^2+^ and Mg^2+^. **e** and **g**, Time courses of currents at −100 mV. Extracellular solution was exchanged from a solution containing 10 mM [Ca^2+^] to 10 mM [Sr^2+^] and 10 mM [Mg^2+^], with 0.02 mM [Ca^2+^] solution between each exchange. **f** and **h**, Averaged peak current densities at −100 mV in each 10 mM divalent ion solution (n=3). The data obtained from cells infected by the control virus were presented in yellow (n=3) and were included in both plots for comparison. Error bars represent bootstrapped 95% confidence intervals. **i-l,** Blockade of Ca^2+^ current of CalC4_24 **(i-j)** and CalC6_3 **(k-l)** by 10 μM La^3+^. **i** and **k**, Time courses of currents at −100 mV recorded in 10 mM [Ca^2+^] solution, followed by addition of 10 μM La^3+^, and finally in 0.02 mM [Ca^2+^] solution. **j** and **l**, I-V relations obtained from a −100 mV to +100 mV ramp protocol before (cyan) and after (purple) addition of 10 μM La^3+^ to the 10 mM [Ca^2+^] solution. Each I-V curve was averaged over three measurements on separate cells, with the baseline current measured in 0.02 mM [Ca^2+^] solution subtracted. The shaded regions in the I-V curves represent bootstrapped 95% confidence intervals.

**Fig 5:**
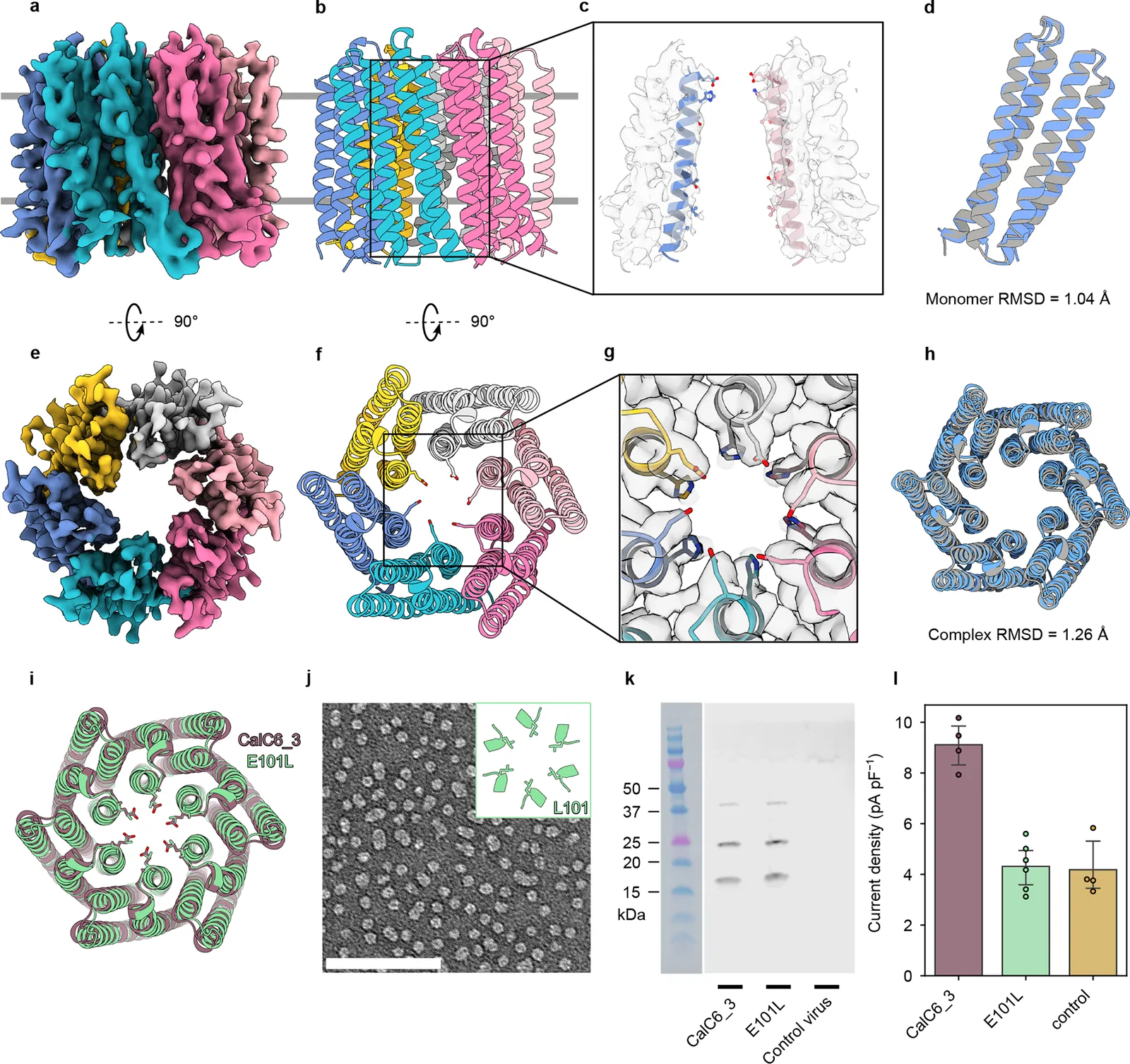
Determination of selectivity filter geometry and function. **a and e,** Side **(a)** and top **(e)** views of sharpened cryo-EM density map of CalC6_3. **b and f**, Structural model of CalC6_3, with the same colors and orientations as the density map in **(a)** and **(e)**, respectively. **c and g,** Close-up views of the pore-lining helices **(c)** and the selectivity filter **(g)**, with the densities shown as gray surfaces contoured at 4 σ. For the side view of pore helices in **(c)** only the opposing monomers were shown for clarity. **d and h,** Structural alignment of the monomer **(d)** and the hexamer **(h)** of the CalC6_3 design model (gray) to the cryo-EM structure (blue), with backbone RMSDs of 1.04 Å and 1.26 Å for the monomer and complex alignment, respectively. **i,** Top view of the AlphaFold-predicted model of the CalC6_3-E101L mutant (in green) overlaid with the predicted model of the wild-type CalC6_3 design (in purple). **j,** Representative ns-EM micrograph of the purified CalC6_3-E101L protein, with a close-up view of the six Leu residues at the selectivity filter site shown on the top right. Scale bar, 100 nm. **k,** Western blot analysis of protein expression of the CalC6_3 design and the E101L mutant in Hi5 cells using an anti-His antibody. **l,** Averaged peak current densities at −100 mV in the 10 mM [Ca^2+^] solution obtained from cells infected by CalC6_3 (purple, n=4), the E101L mutant (green, n=6), and the control virus (yellow, n=4), respectively. Error bars represent bootstrapped 95% confidence intervals around the mean.

## Data Availability

EM maps of CalC6_3 with DHR extensions have been deposited in the Electron Microscopy Data Bank (hexamer: EMD-47340; heptamer: EMD-47356). Cryo-EM structure files of CalC6_3 with DHR extensions have been deposited to the Protein Data Bank (hexamer: 9DZW; heptamer: 9E0H).

## Code availability

Example design scripts are provided in the Github link: https://github.com/ylliu15/2025_Ca_channel. Documentation for RFdiffusion is available at https://github.com/RosettaCommons/RFdiffusion. Documentation for ProteinMPNN is available at https://github.com/dauparas/ProteinMPNN. Documentation for LigandMPNN is available at https://github.com/dauparas/LigandMPNN. AlphaFold2 and AlphaFold3 used for filtering the designs are available at https://github.com/google-deepmind/alphafold and https://alphafoldserver.com/welcome, respectively.
