# Supplementary material for: Bottom-up design of Ca2+ channels from defined selectivity filter geometry
Source: Nature. Author manuscript; Available in PMC 2026 Sep 11. (PMC13560876; doi:10.1038/s41586-025-09646-z)
Supplement: Supplementary file 1 [file NIHMS2202372-supplement-Supplementary_information.pdf]

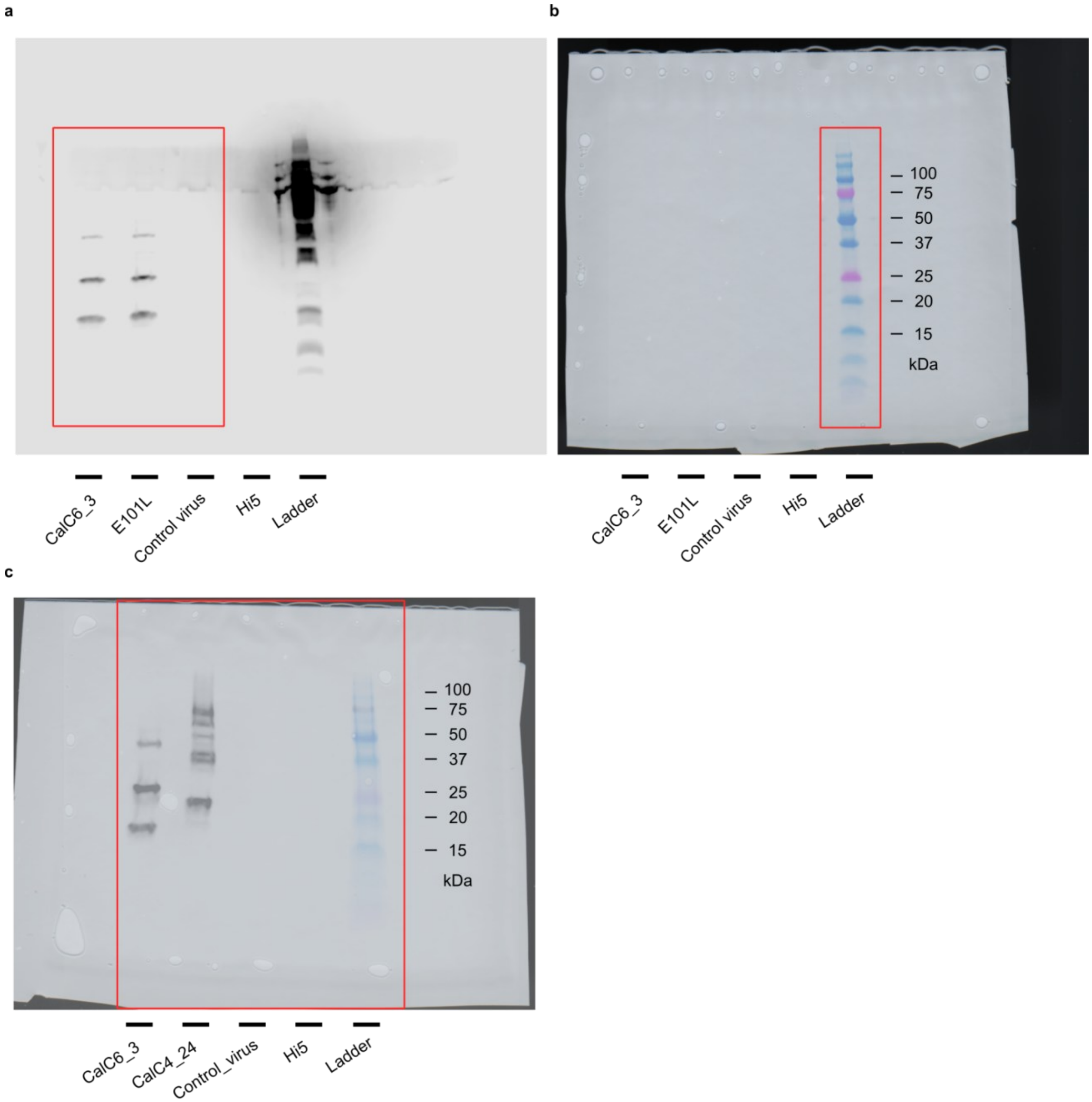

Fig. S1: Western blot source data showing the recombinant expression of the designed channels in Hi5 cells. Red frames indicate the cropped regions shown in the manuscript. **a** and **b** represent the source blots of the result shown in Fig. 5, with chemiluminescence imaging results shown on the left and reflective illumination brightfield imaging results shown on the right. **c** represents the source blot of result shown in Extended Data Fig. 7, with chemiluminescence and reflective illumination brightfield imaging results overlaid using the LI-COR Acquisition Software.

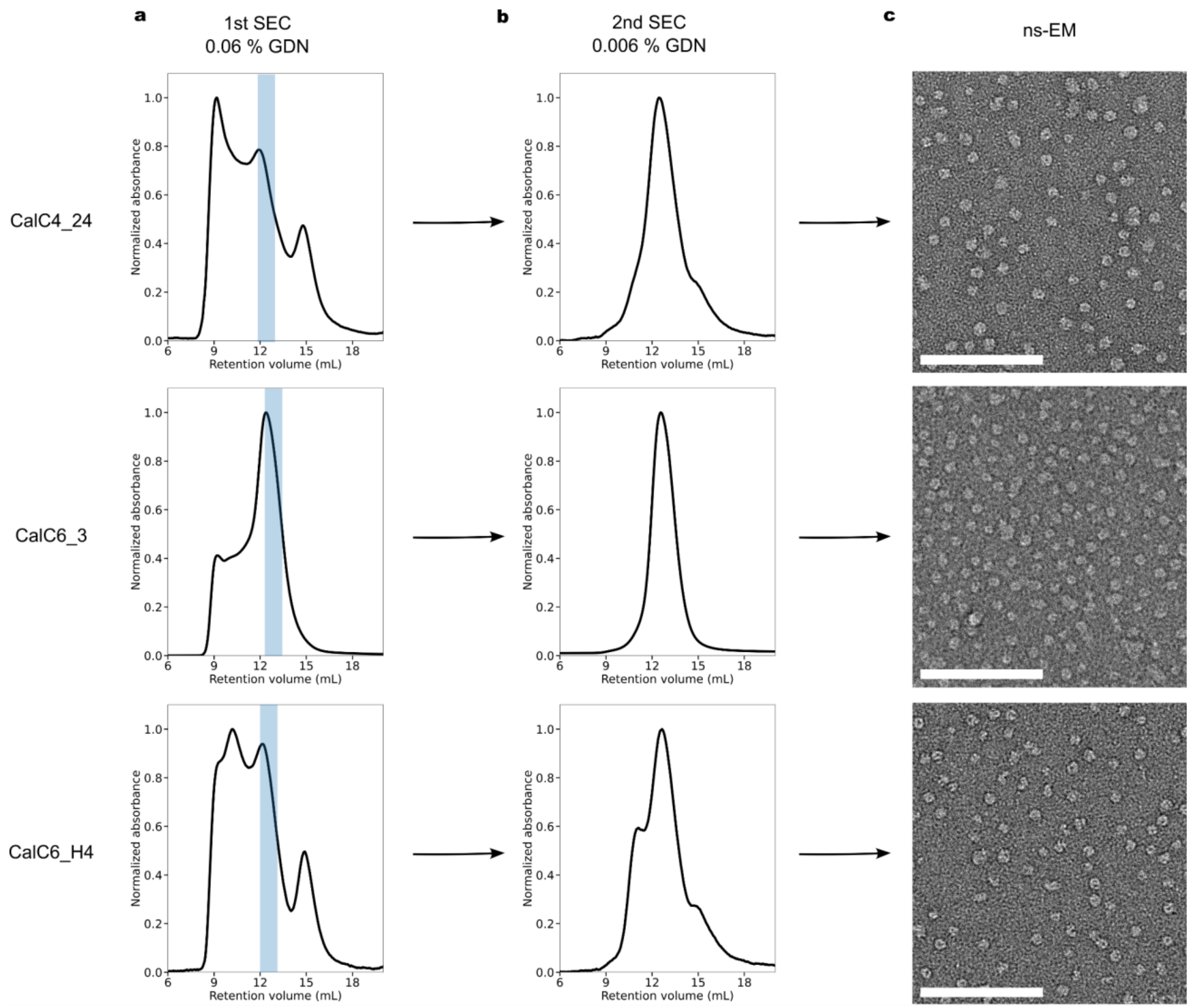

Fig. S2: Illustration of the purification strategy for separating the target protein species with the desired oligomeric states from aggregates. The three designs (CalC4_24, CalC6_3 and CalC6_H4), which were previously shown in Fig. 2, were presented here as examples (top, middle and bottom, respectively). From the first SEC run in the buffer containing 0.06% GDN **(a)**, only the latter portions (blue regions) were taken and were subject to the second SEC run in the buffer containing 0.006% GDN **(b,** also shown in Fig. 2d). Protein samples taken from the more distinct elution peaks were observed to form homogeneous pore-containing particles on ns-EM grids **(c)**. Scale bars, 100 nm.

Table. S1: Amino acid sequences of the designed channels in this study.

Design name	Sequence
**CalC4_1**	MKEQEQLKLKRRILRVIWLALLLTLLAILALIIGLYLVIYASKISTKLALACTFVTC<br>TAAAALVVMFRTIMQEMLRLFESPVVALQAGADMAAAGGIAACTAAAAAGN<br>AAAETKADRRIILYLGIILATTGALIGLLACLFACWFAEEMCEELAKKEPEKREL<br>YEKLAELARWGRLVALLGIIAVIILTIKALYSKSDAEIAVYLTALAVIAAITAILLLLI<br>AFIAGLLVKAY
**CalC4_4**	ARREALRKRLRALGAWAAAAFLLLLLLLLLATLIALYLALKSKSARTVAALTVV<br>LAAFIGPLAEGIRKGLEAIRALYEGDLLGAARGGGEAALAFGVAAASAAGGG<br>ANAALELPKEDAIIALYTGFLLGLVLALLGLVMAWAGIIAGLLGLEETDDPALR<br>RELFEALAEAVIGLITALLALACALLCLAAYYKLLGKEEKADYVLKLAILVVLVV<br>GLLLLWLFAAFARELAAE
**CalC4_24**	EKEEEARKRLTLLAVWMLILDTVLKILLIVLSIIATYIIIRLGNAELLIGMLLGEAL<br>TNAALIGADAKFGEAIKAMLEGNGFETAEALAATAQAYATAGLLGAAGYCN<br>AALELPKEDAVYLIYFGALVALILLLAALAAAWAARLAAEIEIELLPEEEAKKKE<br>IELLVRLRGVLSAIADVAILIFRLLAVAYMLEGRAKEARYCFLLSLILAIVTIVLII<br>VLTWVIIRKILEA
**CalC6_3**	AELRERLLRAARWILLLGLLVLVGFVVLAYLERSPLIRAFVLSAGVVLVALFA<br>AALAWLYLAAALLGRSPLLALVALALGLITLAAASAAMAATFAHLLLEAPPEY<br>REAMLYVFGIAVLIVGLLLLGLVWLLEEALEALLEE
**CalC6_7**	EELRKRLVELAEWVLGVGGLWLVVFGVLAYLEKSTYVAGFTLAAGLALAAIL<br>AIAYAGLALAAVKLEKSEEEAAEVLATTVTAVATAISTAAATAAALLAEAPPE<br>YQPALLYIFVVAAVRALGLLGVLVWLAREFIEELLKK
**CalC6_H1**	ATERLRRQMEWLIIVGVLFFLILILLLLYLMLRADPFTRLVLTAALAMAVAALIV<br>FMAHALKARLARDDVVAALDHLLAGLEAAALLLAAMAAFLASMMLELKDES<br>YIPSVIYIILLGIIGLLVLLLLTAWLVDELLRERAA
**CalC6_H4**	SIKDKLLLLIIWVALLVLLLLIALLIAVSYLLKPLPTLAALFATQLFAIATVVFALFA<br>QAVLKLLSGKDPLEALDLVLAAIATCASSLAALALTLTLLLLELGKEDLALYVL<br>TLLTIATLLLLILLIVFAWVAVKLLEEAKK
**CalC6_H13**	SELEREVLRLLWVFLLVLLLIALVLAAAVYFVLRVDLVLAIALVAFLAALVVIGLG<br>RAVLALIRGDHLAAIDALVAALEALALLLALLGAHLATLLLEKEEERRKIAPYLLI<br>VVGALLILLALWLLAAVLRWLRRQ
**CalC6_H21**	SRERLNKMLRWLGLVALLLLLALIAYLLYLIRQANPRMQLVLTAALAMTVAAL<br>VVTTNHFLKAFLKHEDIEEAIEEVLAGLEATALLLALMAAFLAAMMLELKDES<br>FIPQVIYLLLLGILGLLVVLLLLAWLVGEYLKELES

Table. S2: Top structural alignment results for CalC4_24 identified by Foldseek-Multimer sorted by tTM score.

Database: PDB100						
PDB ID	Seq. Id.	Aln. Length	Prob.	E-value	qTM	tTM
3zci	12.3	194	0.02	45.7	0.07	0.35
6zpp	11.4	140	0.1	9.03	0.06	0.35
6w5q	17.4	126	0.1	8.23	0.04	0.32
3fwv	14.6	116	0.08	19.9	0.04	0.28
2nc9	16.3	92	0.06	19	0.03	0.23
7vg5	14.1	155	0.14	12.5	0.09	0.21
7vg4	11.7	188	0.08	11.8	0.08	0.2
2fji	7	227	0.08	11.9	0.08	0.18
5fzr	13.8	130	0.14	5.42	0.04	0.16
6ak3	10	249	0.05	17.3	0.09	0.15
Database: BFMD						
Accession codes	Seq. Id.	Aln. Length	Prob.	E-value	qTM	tTM
ProtVar_Q04917_Q15843	12.2	180	0.08	11.2	0.07	0.2
ProtVar_Q04917_Q9BV40	11.6	180	0.09	10.7	0.07	0.19
ProtVar_P06454_Q04917	11.6	180	0.1	9.26	0.07	0.19
ProtVar_Q04917_Q9NVL1-2	10.6	188	0.09	9.16	0.07	0.18
ProtVar_Q04917_Q96B36-2	11.6	181	0.08	10.5	0.07	0.18
ProtVar_P62263_Q04917	11.6	180	0.08	12.2	0.07	0.17
ProtVar_P03950_Q04917	11.6	180	0.08	11.7	0.07	0.17
ProtVar_Q04917_Q9UHV9	12.2	180	0.08	11.7	0.07	0.17
ProtVar_Q04917_Q9NVJ2	11.6	180	0.08	11.6	0.07	0.16
ProtVar_O60565_Q04917	10.9	182	0.09	11.7	0.07	0.16

Table. S3: Top structural alignment results for CalC6_3 identified by Foldseek-Multimer sorted by tTM score.

Database: PDB100						
PDB ID	Seq. Id.	Aln. Length	Prob.	E-value	qTM	tTM
8d9p	12.9	162	0.02	100	0.11	0.48
8d9o	11.1	162	0.06	47.5	0.1	0.46
5vju	11.1	171	0.08	29.9	0.09	0.41
2cj8	10.9	119	0.03	58.7	0.07	0.4
5vjt	9.1	174	0.11	17.5	0.09	0.4
1x90	11.5	130	0.04	36.5	0.07	0.39
5vjs	11.6	171	0.05	40.6	0.09	0.39
6z0c	12.5	167	0.08	17.4	0.08	0.36
8dt0	14.1	141	0.02	38.8	0.05	0.33
1t8b	8.9	123	0.03	50.1	0.07	0.31
Database: BFMD						
Accession codes	Seq. Id.	Aln. Length	Prob.	E-value	qTM	tTM
ProtVar_Q8N5M9_R4GN61	13.2	166	0.01	103	0.11	0.41
ModelArchive_ma-fu8t5	12.7	118	0.08	20.6	0.07	0.4
ProtVar_Q8N5M9_Q8WVX3	13.2	158	0.01	109	0.1	0.34
ProtVar_Q8N5M9_Q9Y6X1	14.2	154	0.01	89	0.09	0.32
ProtVar_Q8N5M9_Q96E16	13.2	158	0.01	122	0.1	0.29
ProtVar_B2RUZ4_Q8N5M9	14.2	154	0	174	0.09	0.29
ProtVar_Q8N5M9_Q8NHW4	14.2	154	0.01	103	0.09	0.29
ProtVar_O43653_Q8N5M9	14.1	162	0.01	127	0.1	0.28
ProtVar_Q8N5M9_Q9BU79	13.2	158	0.01	141	0.1	0.28
ProtVar_A2RRL7_P21145	12.7	133	0.03	49.7	0.09	0.28

Table. S4: Top structural alignment results for CalC6_H4 identified by Foldseek-Multimer sorted by tTM score.

Database: PDB100						
PDB ID	Seq. Id.	Aln. Length	Prob.	E-value	qTM	tTM
6z0c	11.6	163	0.03	68.7	0.1	0.41
1yo7	11.1	144	0.03	41.1	0.06	0.4
7dgu	24.5	102	0.38	0.96	0.04	0.34
1few	14.1	141	0.01	52.9	0.09	0.22
3hiu	13.2	121	0.03	44.8	0.07	0.2
4eru	15.6	134	0.02	58.9	0.07	0.2
8e2j	9.3	150	0	147	0.08	0.2
2gs4	19.7	81	0.01	121	0.07	0.19
2gyq	19.7	71	0.04	56	0.07	0.18
1g73	11.1	126	0.01	68.7	0.05	0.16
Database: BFMD						
Accession codes	Seq. Id.	Aln. Length	Prob.	E-value	qTM	tTM
ProtVar_O95214_Q13021	17	141	0.07	18.7	0.08	0.24
ProtVar_Q8N8F7_Q96HE8	19.2	130	0.21	9.35	0.09	0.22
LevyLab_P32842_V1_3_relaxed	12.5	127	0	138	0.08	0.22
ProtVar_P63279_Q13021	17	141	0.04	34.5	0.08	0.22
ProtVar_Q5TGU0_Q969K7	12.6	182	0	197	0.1	0.21
ProtVar_Q96HJ5_Q9NV12	17.7	152	0.01	112	0.1	0.21
ProtVar_Q13021_Q9NQG1	15.3	111	0.04	26.8	0.06	0.21
ProtVar_Q9NR28_Q9Y5J9	9.2	151	0.01	107	0.08	0.21
ProtVar_Q04941_Q9BQA9	13.2	113	0	125	0.08	0.19
ProtVar_P09693_Q969S6	15.8	82	0.06	35.9	0.07	0.19
